# Archaic humans in the Middle Palaeolithic Levant conducted planned and selective intercepts of aurochs, but not mass hunting

**DOI:** 10.1038/s41598-025-26274-9

**Published:** 2025-11-26

**Authors:** Reuven Yeshurun, Gideon Hartman, Hila May, Florent Rivals, Kathryn M. Crater Gershtein, Chen Zeigen, Yossi Zaidner

**Affiliations:** 1 School of Archaeology and Maritime Cultures, Zinman Institute of Archaeology, University of Haifa, Mt. Carmel, 3103301 Haifa, Israel; 2 Department of Anthropology, University of Connecticut, 354 Mansfield Road, Unit 1176, Storrs, CT 06269 USA; 3 Department of Anatomy and Anthropology, Gray Faculty of Medical and Health Sciences , Tel Aviv University, Tel Aviv, Israel; 4 The Shmunis Family Anthropology Institute, The Dan David Center for Human Evolution and Biohistory Research, Gray Faculty of Medical and Health Sciences, Tel Aviv University, Tel Aviv, Israel; 5 ICREA (Institució Catalana de Recerca i Estudis Avançats), Barcelona, Spain; 6 Institut Català de Paleoecologia Humana i Evolució Social (IPHES-CERCA), Tarragona, Spain; 7 Departament d’Història i Història de l’Art, Universitat Rovira i Virgili, Tarragona, Spain; 8 Institute of Archaeology, The Hebrew University of Jerusalem, 9190501 Jerusalem, Israel

**Keywords:** Middle Palaeolithic, Zooarchaeology, Isotope analysis, Dental wear, Projectile impact marks, *Bos primigenius*, Anthropology, Archaeology

## Abstract

While archaeologically challenging, determining whether hominins practised mass hunting before ca. 50,000 years ago is crucial for demonstrating intergroup communication and cooperation. The premise is that killing and processing several herd animals in a single event implicates planning, food sharing, and aggregation at a scale greater than most other Palaeolithic activities. Here, we focus on Unit III in the deep Middle Palaeolithic deposits of the Nesher Ramla karst depression (~ 120,000 years ago) in Israel, an early contact area of archaic and modern humans. Numerous aurochs (*Bos primigenius*) remains were found in this thin, temporally constrained stratigraphic unit, featuring signs of human butchery and consumption. An aurochs tibia displayed an embedded flint chip enveloped by bone remodelling, consisting unique evidence of recapture. We apply ageing, sexing, tooth wear and isotopic techniques to test the hypothesis that this assemblage represents mass hunting events but conclude that the evidence agrees better with multiple isolated, planned, and selective hunting and processing episodes. Thus, our results lend support to the commonly accepted view that Middle Palaeolithic archaic humans lived in small, dispersed, and disconnected groups, which might have been a disadvantage when faced with the sympatric modern humans.

**Supplementary Information:**

The online version contains supplementary material available at 10.1038/s41598-025-26274-9.

## Introduction

The current paper draws on a dense and constrained deposit of bone and lithic finds in primary deposition at the open-air site of Nesher Ramla to explicitly test the possibility of mass hunting during the Middle Palaeolithic (MP) period in the Levant. In doing so, it seeks to engage the widespread hypothesis that archaic human populations in Eurasia (Neanderthals and their contemporaries) consisted of small, fragmented groups that had little contact among themselves^1–7^, a structural feature that amounted to an evolutionary disadvantage when faced with the more numerous or better interconnected modern human groups^8,9^. Presumably, higher population connectivity shores up group resilience and accelerates cultural evolution^1,10^. Accordingly, if we can demonstrate that archaic populations engaged in large-scale events, which required coordination, inter-group interaction, and knowledge exchange^11,12^, we can also falsify the social hypothesis of their exclusion.

Mass hunting, pertaining to the simultaneous killing and subsequent processing of several herd animals, may be a case in point because it may involve high levels of cooperation and communication among numerous people from different bands over several days, sometimes longer. As such, it is an important archaeological proxy of large-scale cooperation^13,14^. However, the archaeological criteria for identifying mass hunting events are challenging. The faunal assemblage must feature a threshold number of prey bones and individuals of the same gregarious species, be convincingly anthropogenic, and feature a single mortality event^15,16^. The age and sex profiles should correspond to those of a living herd, indicating that no selection was applied in the hunt. They should also support the possibility that all animals died in the same season^17–19^, and carcass exploitation needs to be demonstrably relaxed and geared towards preferred cuts or fat-rich individuals^15,17,20^. Based on these criteria, some iconic accumulations of ungulate remains in the European late Lower and Middle Palaeolithic record had been interpreted as mass hunting episodes^21–24^, debated^25,26^, or disproved as such^27,28^. However, in the MP Levant, when and where archaic and modern humans repeatedly encountered each other^29^, no such investigation has been conducted, largely because large and sufficiently preserved archaeofaunal samples were seldom recovered from open-air sites. Upon the transport of processed carcass parts or filleted meat cuts and fat products to “base camps”, the mass hunting signal is usually lost forever; one needs to look into previous links in the chain to find clues to the organization of the hunt^21^. With one exception coming from the Syrian steppe^30^, no sites in the Levant possessed a large, anthropogenic, and stratigraphically well-constrained deposition of large ungulates, before the discovery of the present case-study.

**Fig. 1:**
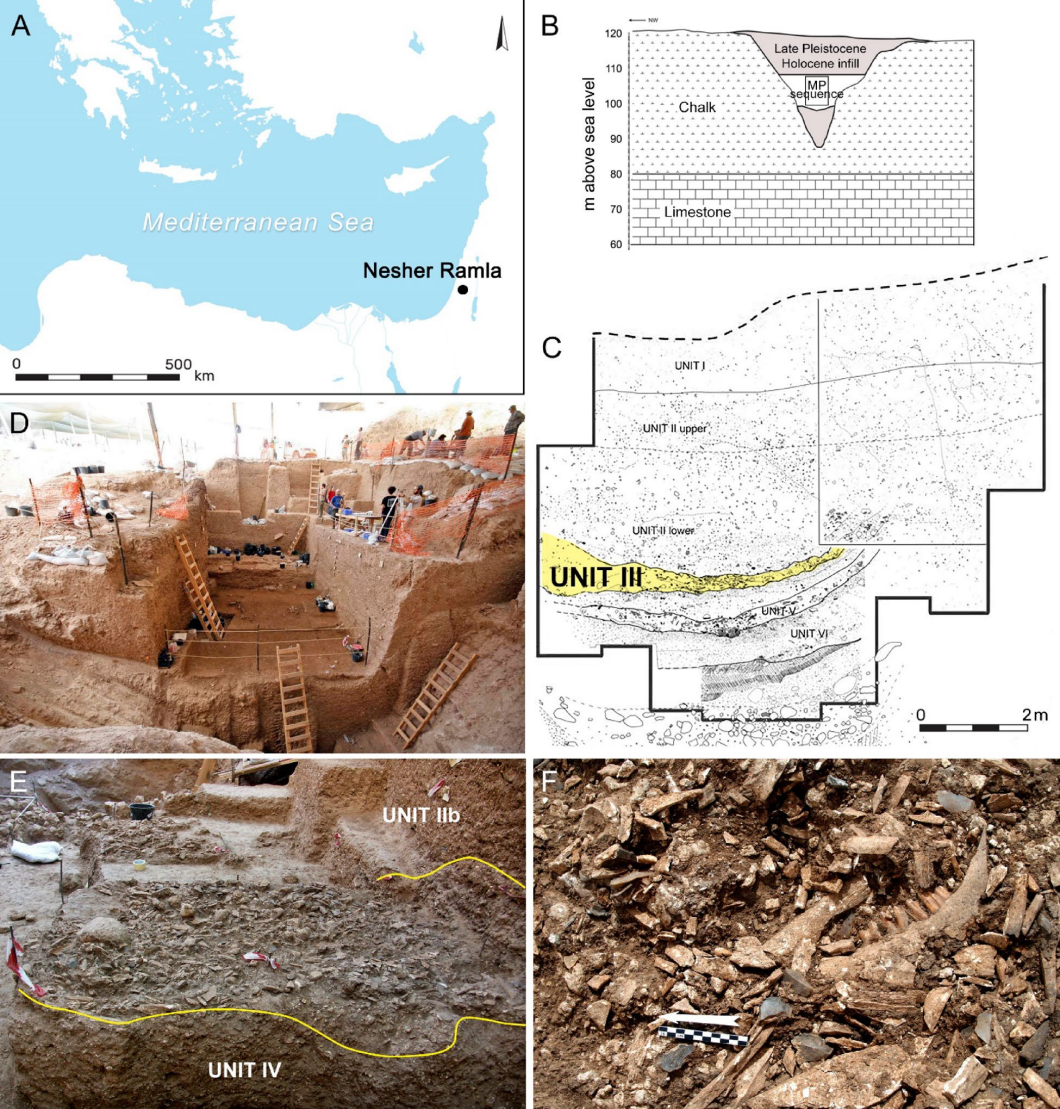
The Nesher Ramla site and its Unit III. **(A)** Site location. (**B)** Geological section of the karst depression (the MP sequence is marked). (**C)** Stratigraphic section of the MP sequence (Unit III is outlined in yellow). (**D)** General view of the excavation, looking west. (**E–F)** Views of Unit III during excavation.

Unit III in Nesher Ramla (henceforth NR III; Fig. 1) is the only comparable case recorded to date, comprising a large, anthropogenic, and well-constrained deposit of large-bodied ungulates (predominantly aurochs, *Bos primigenius*). Our zooarchaeological study of NR III^31^ established that it is a well-preserved assemblage, dominated by aurochs, and deposited by humans. We further interpreted this accumulation as a specialized camp for butchery and processing of the aurochs that were hunted in close proximity, and used the archaeological evidence to argue that entire human groups were present, albeit for short-term occupation/s. The issue of whether hunting was practiced selectively or as a mass event (or a few such events) remained unexplored, due to the limited age and sex information and the absence of supporting bioarcheological information in that study. In the current paper, we test the hypothesis that this deposit derives from a mass hunting event. We contextualise the aurochs abundances against the regional record, and present comprehensive new data on the aurochs herd structure to determine whether selective hunting was practised, dental microwear variability to explore matters of seasonality, and dental stable isotope variability to test if a single herd was exploited. We also revisit the projectile impact mark presented in the former analysis^31^, and present evidence that it constitutes a unique find, a healed hunting injury, which is pertinent for our case. Ultimately, we will argue that NR III did not host large-scale mass hunting events but a series of small-scale selective hunting and processing activities. By extension, therefore, our analysis of NR III supports the hypothesis that archaic humans featured low-level intergroup communication.

## Results

NR is an open-air site inside a large karst depression in central Israel, in the Mediterranean climate zone (Fig. 1A). The site features an eight-m-thick MP sequence, dated to ca. 140–100 ka, with little cultural change along the way^29,32^. Unit VI of the site yielded Middle Pleistocene *Homo* remains bearing a combination of Neanderthal and archaic features^33^. We assume that at least the site’s Lower Sequence (Units VI–III), which is culturally homogenous and chronologically tight^29,32^, represents the activities of these archaic *Homo* groups. The uppermost deposits (Unit I, hence NR I) are thick, display some lithics but no combustion features, and consist of sporadic faunal remains that resulted from multiple predation events by hominins and carnivores, along with non-predation deaths. Evidence of human butchery is infrequent, and articulated vertebral columns attest to in situ preservation. We interpreted this deposit as a site of opportunistic hunting and scavenging by humans and carnivores, probably targeting ungulates that came to the sinkhole to drink. Therefore, we consider this assemblage representative of the site’s “natural” environment^34^. In marked contrast, Unit III is a distinct, ca. 30 cm-thick primary deposit^32^. It spans ca. 55 m^2^ across the entire depression floor, containing combustion features and abundant lithic and bone finds (Fig. 1). The fauna is dominated by aurochs, but also included a considerable share of equid (*Equus* spp.) and tortoise (*Testudo graeca*) remains. The faunal sample studied by^31^ for taphonomy produced cutmarks, hammerstone-percussion marks, and retouching on 27% of the ungulate specimens, while the occurrence of carnivore bone modifications was negligible (2%). Thus, it is safe to conclude that the fauna of NR III was almost exclusively deposited and modified by humans.

### Taxonomic abundances

**Fig. 2:**
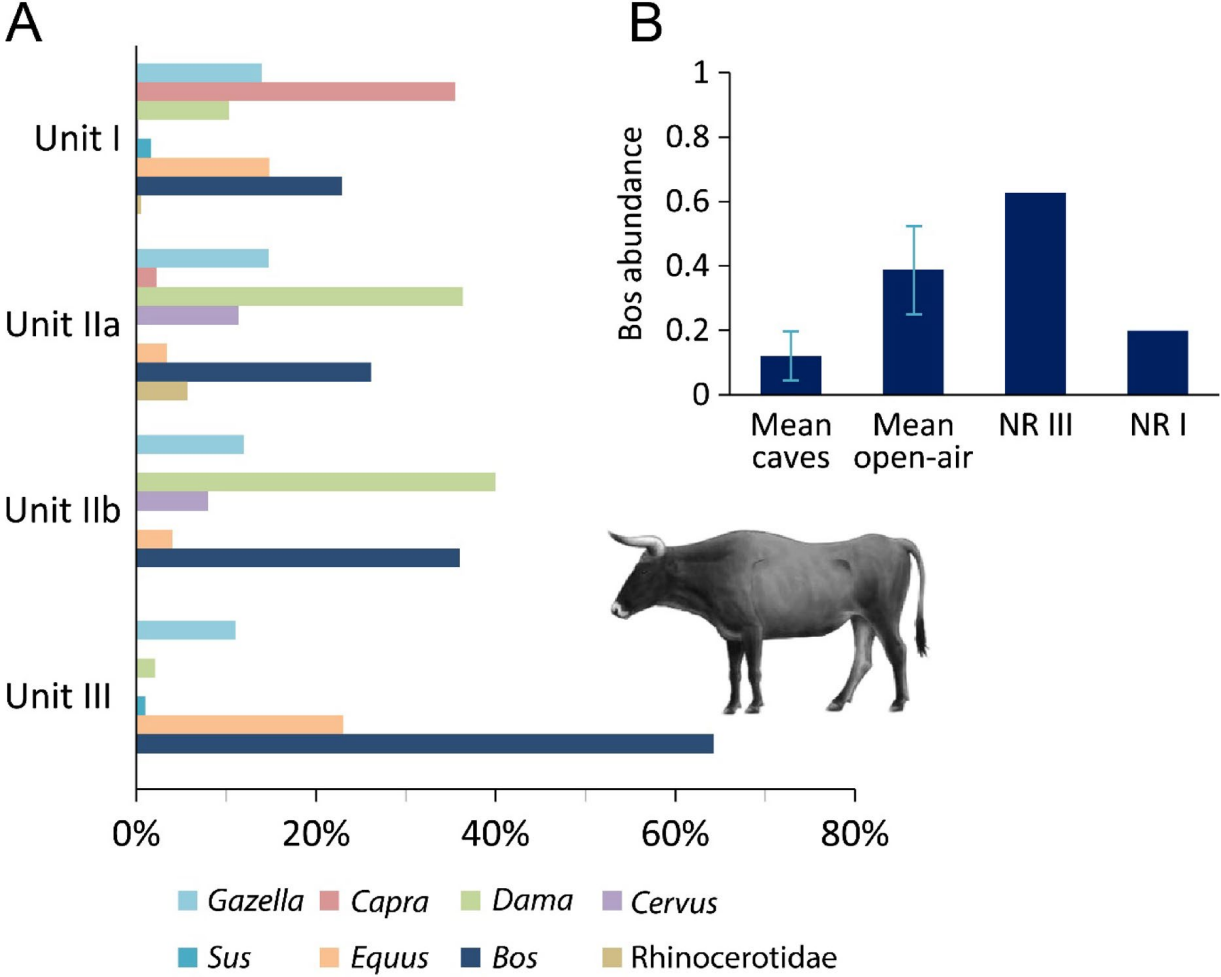
Aurochs abundance in the Nesher Ramla sequence and our study area. (**A**) Ungulate proportional representation along the NR sequence; note the highly uneven ungulate representation of Unit III compared to the naturally-deposited Unit I. (**B**) *Bos* abundance among ungulates at cave sites (n = 9), open-air sites (n = 4; whiskers indicate 1 standard deviation), NR III (anthropogenic), and NR I (mostly natural deposition); See Supplementary Table S1 for data and references.

*Bos* constitutes 64% of the ungulate remains identified to genus at NR III (Table 1), considerably higher than those registered for the largely natural deposition of NR I, which features high ungulate evenness (Fig. 2A), and other Levantine MP sites (Fig. 2B). Other ungulates in the assemblage include equid species (23%) and gazelle (11%). The NR III faunal assemblage produced 229 aurochs specimens, in addition to 258 anatomically-identifiable specimens from the taphonomic sample that were assigned to the large ungulate body-size group (almost exclusively attributed to this species). M_3_ teeth assigned to either side of the jaw indicate that at least 13 individuals are present. Together, the faunal assemblage of this unit includes 478 specimens that are confidently or plausibly attributed to aurochs. While this is not a high number compared to some European bovine-rich sites e.g.,^22,23,25^, the NR III assemblage is much better constrained temporally than most European cases, coming from a 30 cm-thick layer in a finite area that underwent rapid burial.

**Table 1:** Number of identified specimens (NISP) and minimum number of individuals (MNI) for NR III. The left column is based on the taphonomic sample published by Crater Gershtein et al.[31], but some counts differ, due to the reassignment of 81 specimens to the overlying NR IIB. The next column depicts the additional specimens recorded for this study. The total NISP and MNI columns depict the full counts/tallies of ungulate specimens identified to the genus level from the entire NR III unit

	NISP, taphonomic sample	NISP (ungulate genera), rest of assemblage	Total NISP (ungulate genera)	MNI
*Gazella gazella*	13	25	38	3
Small ungulate	17			
*Dama mesopotamica*	2	4	6	1
*Cervus elaphus*		2	2	1
*Sus scrofa*	1		1	1
*Equus* spp.	20	62	82	4
Medium ungulate	92			
*Bos primigenius*	60	169	229	13
Large ungulate (cf. *B. primigenius*)	258			
*Panthera leo*	1			1
*Struthio* sp. (egg-shell)	2			
*Testudo graeca*	229			
*Mauremys* sp.	1			
Total	696	262

NR III provides one of the largest aurochs assemblages in the Levant, in terms of both the tallies and proportional representation (Fig. 2B). Three assemblages in the region may be comparable. In the 1930s, an aurochs-dominated assemblage was collected from the breccia layers of Skhul Cave; however, its aurochs overrepresentation may be due to serious size-related collection and retention biases^35^. High aurochs counts were also recorded in some of Kebara Cave’s MP layers, but none approached the proportional representation seen in our case study^36^. Finally, the extensive excavations at the open-air site of ‘Ein Qashish produced a faunal assemblage, in which aurochs comprise 55%. However, they are considerably fewer than in NR III (NISP = 9–56 per archaeological unit), probably due to slower deposition and unfavourable bone preservation^37^. Thus, no Levantine MP site features the sample size, relative frequency, *and* tightly clustered deposition of aurochs observed at NR III.

### Aurochs herd structure

**Fig. 3:**
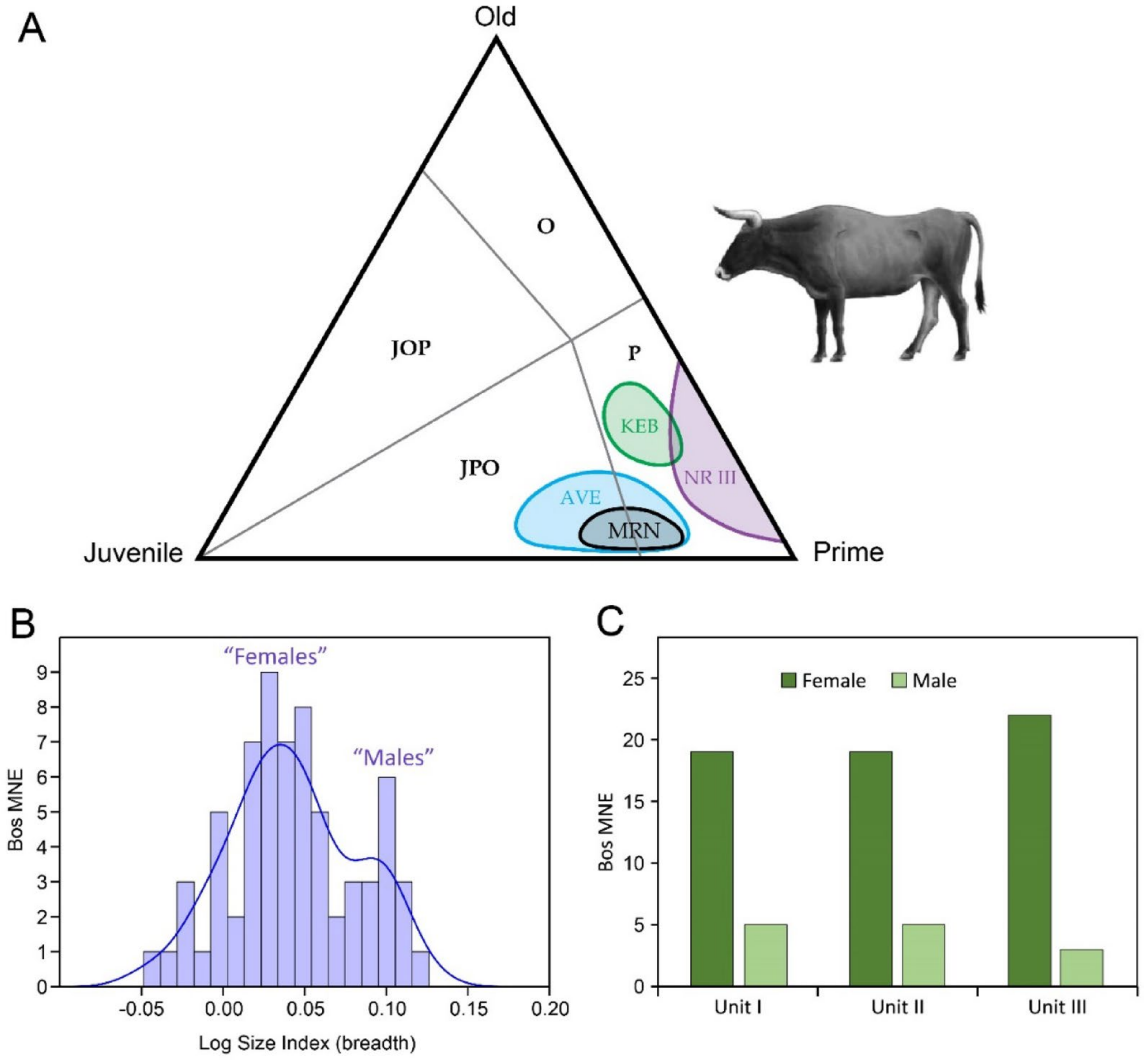
Aurochs age and sex profiles at NR. (A) A triangular graph comparing the aurochs age cohorts (juvenile, prime, and old) in NR III, the MP “residential” camps at Kebara Cave (KEB), the natural death accumulation of Avetrana (AVE), and the bison from the kill site Mauran (MRN; Supplementary Table S3). (**B)** Distribution of aurochs log size index (LSI) values from NR I–III presented as a histogram with kernel density plot; note the bimodal distribution. **C,** Sex proportions of aurochs in NR I–III, based on mixture analysis (Supplementary Table S4).

Bone fusion and dental eruption and wear demonstrate that young and subadult animals are rare or absent in the NR III aurochs sample (Fig. 3A; Supplementary Table S2). Unfused early-fusing elements were not found, and only 7% of the late-fusing bone elements (skeletal elements that fuse at 30–40 months of age, roughly parallel to the P_4_ eruption^38^) have unfused growth plates, indicating that nearly all the animals were fully grown. Similarly, the dental series displays exclusively adult dentitions, mostly of prime-aged individuals and a few old animals (Supplementary Fig. S1 and Supplementary Table S2). Thus, the proportional representation of NR III’s aurochs age cohorts (juvenile, prime, and old) plots in the prime-age (P) zone of the triangular graph, which is incongruent with a living herd profile (Fig. 3A). Although they do not overlap, this pattern is similar to that recorded for the aurochs assemblage in Kebara Cave, which formed via repeated deposition in an MP residential camp. Conversely, the aurochs assemblage from Avetrana (Italy) that formed under natural (non-predation) depositional circumstances, and the bison assemblage from Mauran (France), considered to reflect repeated mass hunting events, lean into the Juvenile > Prime > Old (JPO) zone and feature a living-herd mortality model (Fig. 3A)^20,39^.

The distribution of bone-width measurements, which are sensitive to body mass, is statistically similar throughout NR I–III (Supplementary Figure S2) and produces a bimodal pattern (Fig. 3B). The smaller-sized specimens are interpreted as female and are much more numerous than the larger specimens, which are interpreted as male. A mixture analysis of these data, which assigned each specimen to one of the two sexes, demonstrates that females dominate all NR assemblages and constitute 88% of the NR III population (Fig. 3C). Thus, NR III aurochs assemblage mainly comprises prime-aged cows.

### Dental wear

**Fig. 4:**
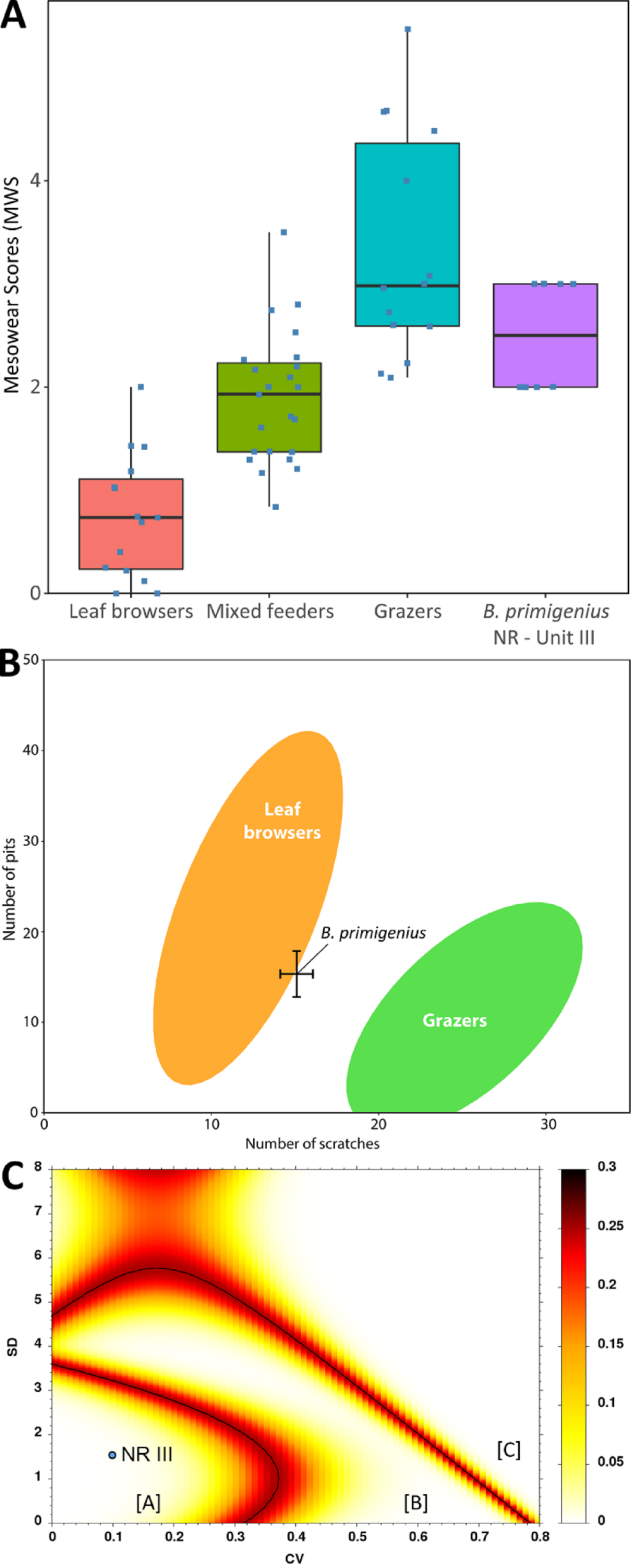
NR III aurochs dental mesowear and microwear patterns. (**A)** Boxplot of the mesowear scores (MWS) for NR III aurochs (right) beside extant leaf browsers, mixed feeders, and grazers^42^; the boxes correspond to the interquartile range, while the bars indicate the minimum and maximum values. (**B)** Bivariate plot positioning the NR III aurochs in terms of their average number of pits and scratches and relative to extant leaf browsers and grazers Gaussian confidence ellipses (after ref.^43^); the error bars for the NR III aurochs correspond to the standard error of the mean (± 1 SEM) for each sample. (**C)** A plot differentiating short events (Region A), lengthy events (Region B), and two separate short events (Region C) of assemblage formation according to microwear variability measured by the convergence of standard deviations (SD) and coefficient of variation (CV) values.

Dental mesowear and microwear analyses provide information on an individual’s diet during the last months-to-years and days-to-weeks of its life, respectively. The dental mesowear of eight individuals indicates a moderately abrasive diet consistent with extant grass-dominated mixed feeders (Fig. 4A; Supplementary Table S5). Dental microwear analysis of 10 individuals found an intermediary number of pits and scratches (Supplementary Fig. S3, Supplementary Table S5), falling just outside the extant leaf browsers’ confidence ellipse (Fig. 4B). Furthermore, many individuals featured large pits and hyper-coarse scratches (Supplementary Fig. S3). Coupled with a high scratch-width score (Supplementary Table S5), these observations indicate a browse-dominated mixed feeding diet consisting of leaves and small twigs at or near the time of death^40^. Assessing the variability of the number of micro-scratches by correlating standard deviation (SD) and coefficient of variation (CV) produced low values consistent with brief, seasonal events (Fig. 4C). Thus, all aurochs seem to have died at more-or-less the same time of year, most likely during the dry summer when green grasses are unavailable but evergreen woody vegetation is abundant^41^.

### Isotope analysis

**Fig. 5:**
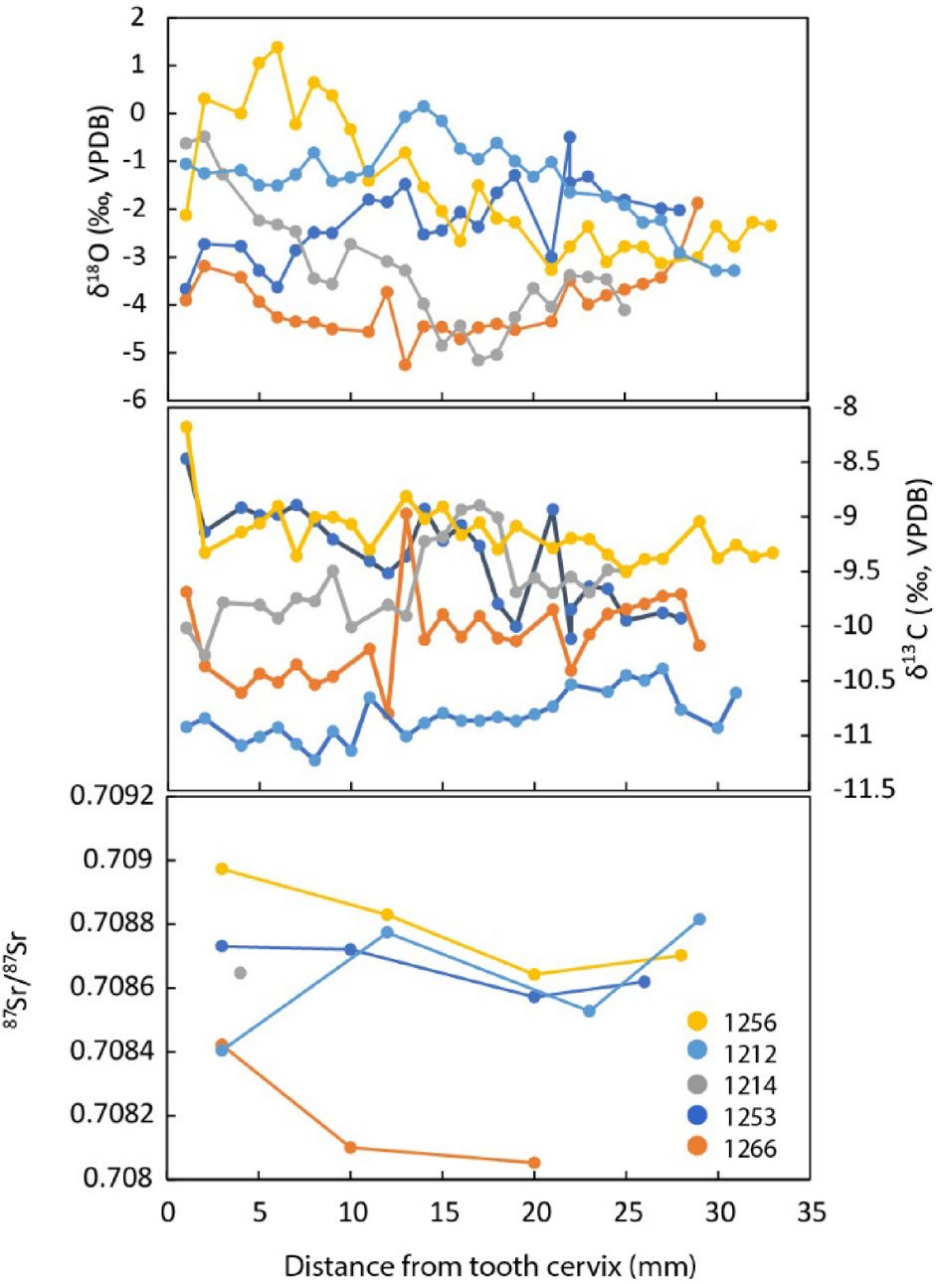
Aurochs dental isotope results. The figure shows incrementally sampled 3rd molars (n = 5). Upper panel showing seasonal oxygen isotope (δ^18^O) values variability; middle panel presents carbon isotope (δ^13^C) values; bottom panel show strontium isotope (^87^Sr/^86^Sr) results (For data summary see Supplementary Table S6 and Supplementary Fig. S4).

Deriving from the enamel of the third mandibular molar, the isotopic signal reflects approximately two years of an individual’s juvenile life stage when still associated with the natal herd^44,45^. Sequential stable (δ^13^C and C) and radiogenic (^87^Sr/^86^Sr) isotope analysis of tooth enamel of five aurochs demonstrates that they originated in different locations and had distinct life histories. Single-factor ANOVA testing of isotopic variables grouped by specimen demonstrates fully differentiated individuals (δ^13^C F(4,129) = 100.91, *p* < 0.0001; δ^18^O F(4,129) = 30.61, *p* < 0.0001; ^87^Sr/^86^Sr F(4,11) = 6.51, *p* < 0.001). Thus, for instance, Individual 1256 spent some time near the Mediterranean coast (^87^Sr/^86^Sr = 0.7090^46^; Fig. 5), and Individual 1266 roamed the Neogene chalk hills surrounding NR and ingested water that originated at relatively high altitudes (^87^Sr/^86^Sr = 0.7080; δ^18^O≈-4‰). Under these circumstances, the NR III aurochs assemblage is unlikely to have derived from the same herd. This is despite the tight stratigraphic clustering and the identification of most aurochs specimens to females (see above).

### A case of recapture?

**Fig. 6:**
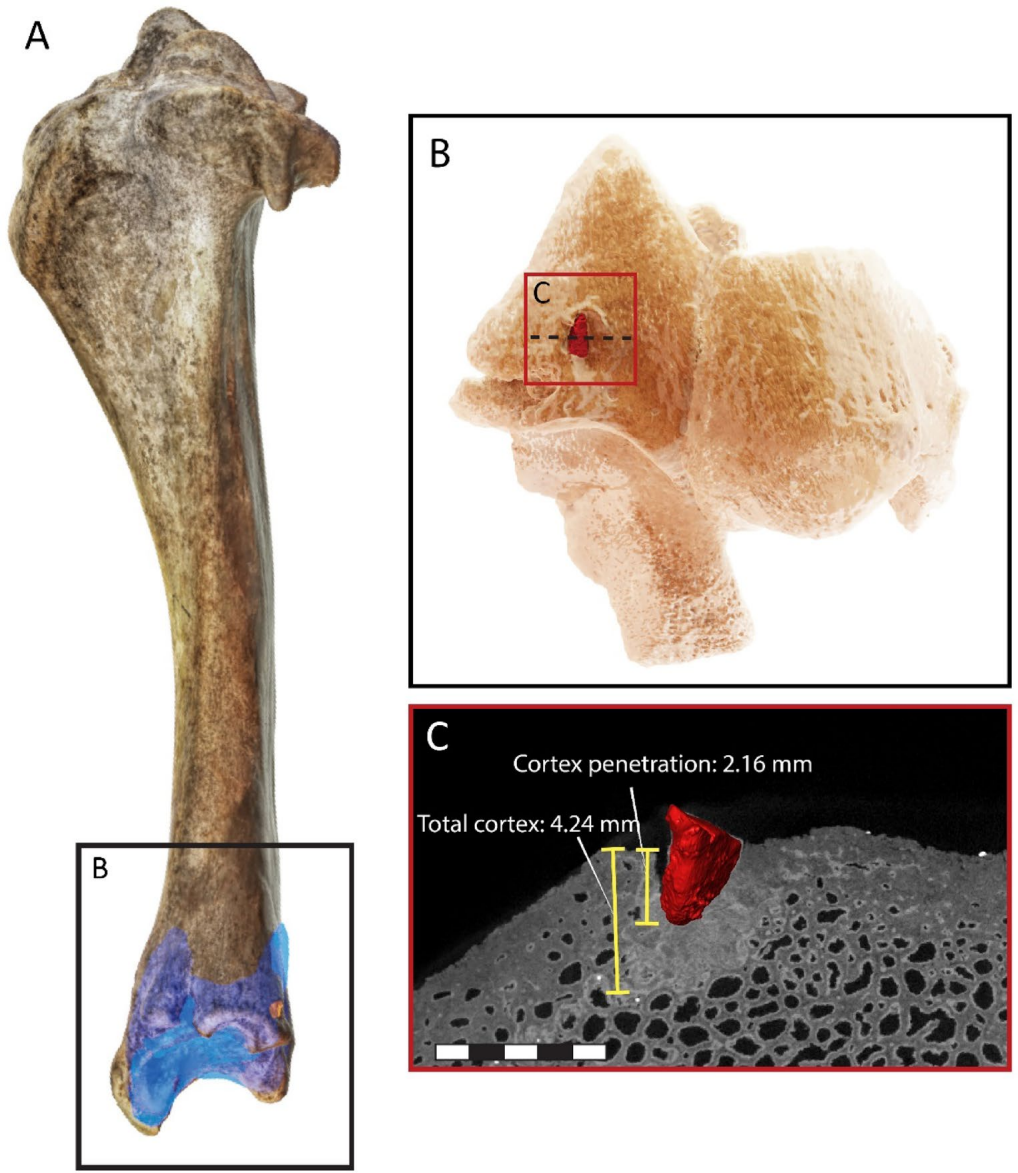
A flint chip embedded in an aurochs distal tibia from NR III. (**A**) Superimposition of the distal tibia fragment on a complete aurochs tibia. (**B)** Surface reconstruction of the distal tibia with the flint chip fragment (in red). The dashed line indicates the location of the bone cross section. **(C)** Cross section of the bone and the embedded flint chip (in 3D); note the bone remodelling around the flint chip.

A notable specimen in the NR III assemblage is a distal left tibia of an adult aurochs with a broken flint chip (6.2 × 3.94 × 3.13 mm) embedded above the lateral malleolus (Fig. 6A; see also ref.^31^). It penetrated the bone posterolaterally (Fig. 6). Micro-CT (µCT) scanning confirmed that the flint item penetrated approximately 2.5 mm into the bone and that new tissue formed around it. The new compact bone was approximately 2 mm thick and sclerotic, corresponding to the compact bone thickness in non-damaged regions (Fig. 6C). This process of new bone formation is likely to have lasted at least four weeks^47–49^. Thus, this specimen belongs to an individual aurochs that escaped a hunt but was recaptured at a later time.

## Discussion

While there is wide agreement that post-50 ka *Homo sapiens* populations engaged in mass hunting^50^, some scholars suggest it had begun as early as 400 ka^23^. Capitalizing on the unique instance of NR III, we hoped to find evidence for mass hunting in the earliest “contact zone” between archaic and modern humans (i.e., the Levant) and thus contribute to this debate. Moreover, we reasoned that procuring such evidence would demonstrate that MP archaic humans exercised considerable intergroup connectivity and established larger effective populations than otherwise believed. Such evidence would counter the prevailing demographic hypothesis that archaic humans could not compete with the communication capacities and larger effective populations of the spreading modern humans. This hypothesis theorizes that demographically inferior populations enjoyed fewer opportunities to interact and generate economic and social innovations^51,52^ and might have been disadvantaged due to interbreeding, allee effects, and stochasticity^53,54^.

Set in a region notable for its human biological diversity during the MP, NR III seemed a particularly fascinating opportunity to engage these issues. While NR yielded anatomically archaic human remains^33^, remains of anatomically modern humans were found in the seemingly contemporaneous deposits in Skhul and Qafzeh Caves and in the earlier MP occupations at Misliya Cave^55^. During the later MP, the region was primarily populated by Neanderthals, who might have repeatedly engaged with African *Homo sapiens* populations^56,57^. Ultimately, however, our analyses and data do not support the hypothesis that NR III hosted mass hunting events, speaking for multiple small-scale hunting events instead.

Drawing on ethnohistoric work and well-documented Holocene archaeological contexts, a series of expectations was formulated for identifying archaeological cases of gregarious ungulate mass hunting^16^. Significantly, the size of the large ungulates precludes the transport of complete carcasses, requiring the establishment of a butchery and processing camp at or very near the place of the kill e.g.,^58,59^. We may expect such camps to be located at or near places with topographic features that disadvantage the animals (i.e., marshes, cliffs, gorges, or pits) and are favourable for mass hunting^21^. These site’s archaeofauna ought to feature a distinctly dominant ungulate species, a mortality profile approximating a living herd, patterns consistent with relaxed carcass processing, indications that hunting took place in a single season, and evidence that all individuals belonged to the same herd^16,27,28^. A spatially and temporally delimited bone deposition where these criteria are met may be interpreted as indicative of mass hunting.

The ethnohistoric literature indicates that mass hunting would have been a suitable—and at times, optimal—hunting tactic for capturing gregarious bovines at higher return rates and reduced risk of failure compared to encounter hunting^14^. Comprising a substantial aurochs bone accumulation in a delimited anthropogenic layer nested within a large karst depression, NR III kindled our hopes to demonstrate that mass hunting was practised here. Some of the evidence we produced during the present study can be taken to support this hypothesis. The abundance of aurochs at NR III greatly exceeds the landscape’s natural availability as embodied by the site’s uppermost unit, NR I^34^. In this unit, aurochs comprised only 23% of the ungulate population, less than half the frequency of the hunted assemblage of NR III. Moreover, the homogeneity of dental microwear patterns could indicate that all the aurochs were hunted during the same time of year when they sustained on a leaf-browsing diet.

On the other hand, other lines of evidence undermined the mass hunting hypothesis. The assemblage’s age profile was one. Aurochs herds consist of females of all ages, calves, and young males, while adult males are more widely dispersed^60^. Thus, a mass hunting site that conforms with a living-herd mortality profile is expected to produce the remains of adult females and young and subadult individuals. The NR III aurochs population does not conform to this pattern. Primarily comprising prime-aged cows, it is too specialized and suggests purposeful selection. Nevertheless, we might note some Holocene bison kill sites dominated by prime-aged females and featuring fewer juveniles than expected. Explanations for this pattern included high 1st-year juvenile mortality in harsh climates^61^ and cows’ higher susceptibility to being caught because they lead the stampeded column^62^. However, these observations and theories do not necessarily apply to aurochs in the mild Mediterranean climate zone. But even if they would, they still could not explain the nearly total absence of juveniles and older subadults in the NR III assemblage.

One might seek to reconcile NR III’s prime-dominated profile with the age distribution of mass hunting via mechanisms of selective processing. Thus, for instance, analyses of several monospecific MP butchery sites in Europe suggest that young animals were often discarded after being killed and left unprocessed^17,20,21^. Some sort of selection may be observable in NR III. Crater Gershtein et al.^31^ demonstrated that the aurochs body-part profile is incomplete and biased in favour of heavily processed meat- and fat-rich elements, suggesting that NR III served as a camp for processing the hunted aurochs. Still, the aurochs skeletal-element profile is much more complete than seen at MP cave deposits in region, many of which were probably base-camps that are further removed from the kill in the hunting-to-discard chaîne opératoire, thus receiving sporadic aurochs skeletal elements (but possibly filleted cuts from the processing sites)^36^. Due to the very large size of the aurochs, transporting multiple large and heavy animal parts into a karst depression only makes sense if they had been killed in close proximity^31^, perhaps because the NR depression was the closest place to finish processing the animals with some level of protection. According to this hypothesis, we may say that, as an initial processing camp, NR III received selected elements of prime individuals from a nearby mass hunting site, while the juvenile individuals were left unprocessed in the field. An alternative to our previous interpretation^31^ is that the NR III inhabitants killed and initially processed the animals in the depression, but extracted the head and toes for intensive fat and hide exploitation elsewhere (e.g.^63^). Under the latter scenario, prime-age cows were selectively hunted, one or few animals each time.

Even stronger evidence against mass hunting derives from stable isotope data. Extracted from the same teeth used for the wear analysis, these data demonstrated heterogeneous seasonal patterning and, by implication, biographies. Previous attempts to harness isotopic methodology to differentiate mass and small-scale hunting events include a single-sample-per-tooth, multiple-isotope ratio analysis of modern pronghorn^64^ and sequential sampling of equid teeth in the Palaeolithic site of Schöningen^27^. Notably, the latter found similar seasonal patterns across various layers, thousands of years apart; thus, patterns which otherwise would have been taken to suggest a single herd emerge as reflections of environmental constraints rather than a uniform herd pattern. In NR III, on the other hand, isotope analysis registered environmental diversity and distinct life histories, making a strong case against the assemblage’s derivation from the same herd. That our specimens are likely to be females strengthens this interpretation. Accounts from the last-surviving European aurochs herds and modern feral cattle populations suggest that female herd structure was stable, while adult bulls roamed more widely and intermittently joined these herds in the mating season^60^. If Levantine MP aurochs behaviour was similar, then we are likely to find uniform life-history signals among females if they come from a single herd.

Lastly, the aurochs tibia with the embedded lithic chip is one of the earliest osteological pieces of evidence of projectile-assisted hunting^65,66^. To the best of our knowledge, the healed injury that indicates a failed hunting event is unique in the Palaeolithic record, with a possible exception in the German Magdalenian^67^ and sporadic later (Early Holocene) parallels^68,69^. The new bone tissue and its thickness not only indicate that the aurochs did not die from the injury but that it persisted for some time after the event^70^. These observations may be taken to suggest that hunters returned to the same place and recaptured the animal, perhaps a familiar individual. However, we cannot rule out the possibility that the herd containing this individual moved considerably across the landscape after the failed hunting attempt and was subsequently captured by a different human group, or that the successful hunter/s belonged to a different group than the ones who inflicted the injury. Regarding the second point, we cautiously suggest that it is less likely that distinct human groups were using the same place to hunt aurochs during a limited time span.

Altogether, the evidence we collected argues against a single-deposition event in NR III or several large-scale hunting events. While the faunal assemblage is aurochs-dominated and the seasonal signature is strong, there is much to point in the other direction: the mortality profile is highly selective and does not reflect a living herd pattern; enamel biogeochemistry is variable and incompatible for a single herd; and at least one individual was recaptured at least several weeks after a failed hunting attempt. Thus, we must reject the hypothesis of mass hunting at NR III.

Europe’s pre-Upper Palaeolithic archaeological record features several monospecific bovine sites, which may have functioned as mass hunting sites. One of the most convincing examples is also the oldest, the 400 ka-old bison bone bed at TD10. 2 (Atapuerca, Spain), which fulfills Lubinski’s^16^ criteria for mass hunting^23^. In the MP, some European open-air sites display numerous human-processed bovine remains that accumulated over relatively short periods, but their attribution to mass hunting is often a matter of debate^18,25,26^. Another mode of large-group animal exploitation in the MP was put forth by Gaudzinski-Windheuser and colleagues^71^. They demonstrated that Neanderthals in the northern European Plain repeatedly hunted adult male straight-tusked elephants during the Eemian. Significantly, these animals were enormous—equivalent in size to several aurochs—and the consumption of just one may have entailed the aggregation of disparate Neanderthal groups. Alternatively, storage was suggested as a means to dealing with the vast amount of animal products in a single hunting episode^71^.

Nothing similar is presently observable in the MP Levant, a long-term interaction zone between archaic and modern humans. Our study demonstrates that archaic hominins in NR carefully planned and repeatedly intercepted female aurochs herds, likely during the summer, purposefully targeting prime-aged cows. It does not support the hypothesis of a large-scale mass hunting event and, by extension, intergroup cooperation and aggregation. Admittedly, no suitable data exists at present to evaluate whether MP (or Early UP) modern humans in the Levant engaged in mass hunting, as no open-air fauna-bearing camps that can be attributed to modern humans are known in the region. Until some evidence for the existence of intergroup or large-group activities is put forth, we may regard Levantine hominins, as being demographically disadvantaged. Our “negative” evidence (i.e., no evidence for aggregations or intergroup events is currently available in the MP Levant) joins multiple “positive” evidence from the Levantine MP showing that MP humans in the Levant predating ca. 60 ka very likely lived in small and relatively disconnected groups, allowing them to hunt the largest available animal on the landscape at will^72^. Under these circumstances, a fragmented population structure would have been an important contributor to their demise or assimilation in the case of a demographically superior population flow onto the Levant.

## Materials and methods

### Zooarchaeology

This study relies on our published zooarchaeology and taphonomy analysis of a sample comprising ca. 40% of NR III’s excavated volume^31^. We employed a standardized procedure that recorded all identifiable mammal remains (other than rodents, insectivores, and bats), all birds other than Passeriformes, and all reptiles. Skeletal-element identification was as inclusive as possible, and each identified specimen was systematically scrutinized for bone-surface modifications and bone fracture patterns. For the full details on these procedures, see^31^. The plots summarizing the taxonomic results were prepared by Microsoft Excel. For the present study, we additionally recorded all the NR III mammal specimens that could be identified to at least the genus level. We explicate our ageing and sexing methods, which were applied to the entire NR III aurochs assemblage.

For ageing, we documented bone fusion data^38^ based on Minimum Number of Elements (MNE), recorded dental eruption and wear for mandibular teeth according to Jones and Sadler’s^73^ age stages (henceforth J-S stages), and measured molar crown heights (see below). Following Stiner^74^, we lumped the dental results into three age classes: juvenile, prime, and old. *Juvenile* corresponds to J-S stages A–D (up to 26–28 months of age); *prime* corresponds to J-S stages E–J (up to ca. 14 years old), and *old* corresponds to J-S stages J–K. The juvenile stage is defined by a present dP_4_ or an unworn M_3_, and the adult stages (i.e., prime and old) are determined and differentiated according to the degree of wear on the M_3_ and M_2_ combined with the cement/enamel junction and the root arch position (Table 2 in^73^).

The prime-old cut-off is often ambiguous because tooth wear patterns are non-linear and feature increasing variability with age. In a study of known-age cattle, the old animals (14 years or more) were assigned to J–S stages K and occasionally J. However, many other animals that were assigned to stage J were considerably younger (Table 1 and Fig. 6 in^73^). Additionally, isolated teeth and incomplete specimens could only be assigned to ranges that span the adult-elderly cut-off (e.g., J–S stage G–J or J–K). In these cases, tooth crown heights (CH) were used to separate younger from older adults.

CH measurements pertain to the minimum distance between the occlusal surface and the base of the crown’s enamel (above the dentine of the root), measured on the buccal aspect on both the anterior and posterior lobes^74^. Because bovid molar crown bases continue to grow for some time after the tooth’s eruption, we followed Gaastra’s^75^ advice to use the time of the root’s initial separation as a baseline for CH measurement. In the *Bos* M_3_, this separation coincides with J-S stages Ec–Ed, approximately 26–36 months of age^73^. In the NR assemblage, a relatively worn tooth of J-S stage Ed produced a CH measure of 56 and 57 mm for the anterior and posterior lobes, respectively. Considering the tooth’s wear, we rounded this measure up and set the baseline for both lobes at 60 mm, acknowledging some (probably inevitable) inaccuracy in our scheme. Following Stiner’s^74^ convention that the prime-old cut-off occurs when half the CH is worn away, we placed this cut-off at a CH of 30 mm. Thus, and notwithstanding the ubiquity of isolated teeth in the NR assemblage, we were able to assign almost all *Bos* M_3_s to one of the three age cohorts using a combination of J-S wear stages and (secondly) crown heights. The proportional abundance of the three age cohorts was plotted on a triangular graph according to the mortality models suggested by Discamps and Costamagno^19^ for extant wild bovines. Ninety-five percent confidence intervals were drawn around the data points, using the program provided by^76^, the locations of which were examined relative to the mortality models and their degree of overlap.

Sexing drew on aurochs’ sexual dimorphism to assess the number of male and female specimens in the represented population. Bone measurements were taken following von den Driesch^77^. To obtain a sufficiently large sample size, we converted all breadth and width measurements (one measurement per specimen) to log size indexes (LSI; ref.^78^), using the early Holocene *Bos primigenius* cow from Denmark as standard^79,80^. Assuming the larger bone specimens are male and the smaller ones are female, as confirmed by postcranial width and breadth measurements of modern, known-sex cattle^81,82^ and by molecular confirmation of osteologically sexed archaeological specimens^83^, we applied a mixture analysis to assign each specimen to either group. Mixture analysis is a maximum-likelihood method that builds on a pooled univariate sample for estimating the parameters of two or more univariate normal distributions^84^. We performed these statistical tests and output plots using PAST 4.03 software^85^.

### Dental wear

Dental mesowear and microwear analyses were performed by a single observer (FR) on ten aurochs left M_3_s from NR III. We used only this tooth in order to avoid duplicating individuals. The occlusal surfaces of the teeth were first cleaned with acetone and 96% ethanol; we then produced a mould using high-resolution silicone (Heraeus Provil novo medium regular set), from which a positive cast of transparent epoxy resin (C.P. Quimica 1060/A and 1585/B) was formed.

Mesowear was scored according to the system developed by Milhbachler et al.^86^ and Rivals et al.^42^. The dental cusps were classified according to their relief and shape into seven integer numerical categories, where 0 indicates high relief and sharp cusps, and 6 indicates blunt cusps (no relief). Individual cusp scores were averaged to obtain the mesowear score for each tooth.

Microwear was analyzed on the M_3_’s protoconid according to the protocol established by Solounias and Semprebon^43^. Microwear features—pits and scratches—were counted over an area of 0.16 mm^2^, using a Zeiss Stemi 2000C stereomicroscope at a magnification of 35 × . Pits are rounded scars that can be small (bright) or large (darker); scratches are elongated scars that can be divided into fine, coarse, and hyper-coarse, depending on their width. Each specimen was assigned a scratch width score (SWS) that ranges from 0 to 2. A score of 0 designates a surface predominantly featuring fine scratches; a score of 1 pertains to a mixture of fine and coarse scratches, and a score of 2 indicates a surface primarily consisting of coarse scratches. The presence or absence of cross-scratches (i.e., scratches that are oriented differently than the main axis of scratches) was also recorded.

The duration of the assemblage’s deposition was estimated according to the variability in the number of scratches, which was quantified by cross-referencing the standard deviation (SD) and the coefficient of variation (CV)^87^. The premise is that variability in the number of scratches is a function of the diet, which depends on the season. On these grounds, we may differentiate three types of accumulations: (A) accumulation within a specific season (single or repeated), (B) accumulation over more than one season, and (C) accumulation in two non-consecutive seasons. The analysis and output plots were performed in R^88^ using the MicrowearBivaR script^89^

### Isotope analysis

The cattle teeth were sampled and processed in the stable isotope preparation laboratory of GH, at the University of Connecticut. Teeth surfaces were first cleaned with a sand blaster using alumina powder. Tooth Enamel was sampled exclusively from M_3_ teeth with a micromilling system and a diamond-coated drill bit. Sample sequences were drilled at 1-mm intervals, starting from the crown cervix. For the purpose of surface cleaning, the upper 5 µm were drilled and discarded. Individual powdered samples weighing 6–11 mg were collected into 0.6 ml Eppendorf tubes and digested for four hours in 0.5N acetic acid to remove diagenetic carbonates that might have adhered to the tooth’s surface. Samples were then washed with deionized water (*dd*H_2_O) until they were neutralized and dried in a desiccating oven at 50 °C for at least 24 h.

^18^O and δ^13^C of carbonates were measured at the Environmental Isotope Laboratory at the University of Arizona, using an automated carbonate preparation device (KIEL-III) coupled with a gas-ratio mass spectrometer (Finnigan MAT 252). Powdered samples were reacted with dehydrated phosphoric acid under vacuum at 70 °C. The isotope ratio measurement was calibrated based on repeated measurements of NBS-19 and NBS-18 reference samples; the resulting precision was ± 0.1 ‰ for δ^18^O and ± 0.08‰ for δ^13^C (1 sigma).

Three to four samples from each tooth were analyzed for the ^87^Sr/^86^Sr ratio at the clean lab facilities of the Yale Geochemistry and Chronology Center. Samples were transferred to Savillex beakers, digested for 12 h in 2 ml of 15.6N (70%) nitric acid (HNO_3_), and left to evaporate at 150 °C until completely dry. The samples were then re-dissolved in 0.5 ml 8N HNO_3_ and loaded into 1 ml columns containing preconditioned 100–150 µm bead Sr-Spec resin (EiChrom Technologies, CAT# Sr-B100-A). Samples were washed with 6 ml of 8N HNO_3_, which was followed by 4 ml of 3N HNO_3_. Next, Sr was eluted with 4 ml of ddH_2_O, dried, and re-dissolved in 5% HNO_3_ to dilute the solution so that it would give ^88^Sr signal intensities of 45–50 V. Solutions were measured using a Thermo Fisher Neptune multicollector plasma ionization mass spectrometer (ICP-MS). Corrections were made to account for the presence of krypton (^87^Kr) in the carrier gas (Ar), for trace rubidium (^87^Rb), and for instrumental mass bias. The results were externally corrected to the NBS-987 standard. The mean ^87^Sr/^86^Sr ratio for the standards run during the analysis was 0.71034 ± 0.00003 (2 sigma; n = 46). Sr concentrations (ppm) in the measured samples were calculated for each run based on the regression equation between ^88^Sr signal intensities (V) measured for four NBS-987 standards and their known concentrations (10, 50, 75, 100 ppb). The results were analyzed and plotted using Excel Data Analysis Package, and image processing was conducted using Adobe Illustrator.

### Embedded lithic analysis

Although rare, every once in a while, the Palaeolithic archaeological record produced an animal bone with an embedded lithic item^65,66^. Experiments have shown that such specimens result from projectile impact or lithic retouching with bone. Marks associated with the embedded item and its anatomical context usually enable researchers to distinguish between the two^90^. No diagnostic retouching marks were observed on the bone in question, an aurochs distal tibia fragment^31^.

The bone was µCT scanned twice using a microfocus X-ray computed tomography system (XT H 225 ST, Nikon Metrology NV, Leuven, Belgium) at the Shmunis Family Anthropology Institute, the Dan David Center for Human Evolution and Biohistory Research, Faculty of Medical and Health Sciences, Tel Aviv University, using a 225 kV 225 W reflection target and the following scan parameters: first scan, 25 µm, 180 kV, 156 µA; second scan, 15 µm, 180 kV, 156 µA.

## Supplementary Information

Below is the link to the electronic supplementary material.

Supplementary Material 1.

## Data Availability

All data are available in the main text or the supplementary materials.
