# Supplementary material for: Archaic humans in the Middle Palaeolithic Levant conducted planned and selective intercepts of aurochs, but not mass hunting
Source: Sci Rep. 2025 Nov 26;15:42237. doi: 10.1038/s41598-025-26274-9 (PMC12658198; doi:10.1038/s41598-025-26274-9)
Supplement: Supplementary file 1 — Supplementary Material 1. [file 41598_2025_26274_MOESM1_ESM.docx]

Supplementary Materials for

**Archaic humans in the Middle Palaeolithic Levant conducted planned and selective intercepts of aurochs, but not mass hunting**

Reuven Yeshurun^*^, Gideon Hartman, Hila May, Florent Rivals, Kathryn M. Crater Gershtein, Chen Zeigen, Yossi Zaidner

*Corresponding author. Email: [ryeshuru@research.haifa.ac.il](mailto:ryeshuru@research.haifa.ac.il)

**This PDF file includes:**

Figs. S1 to S4

Tables S1 to S6


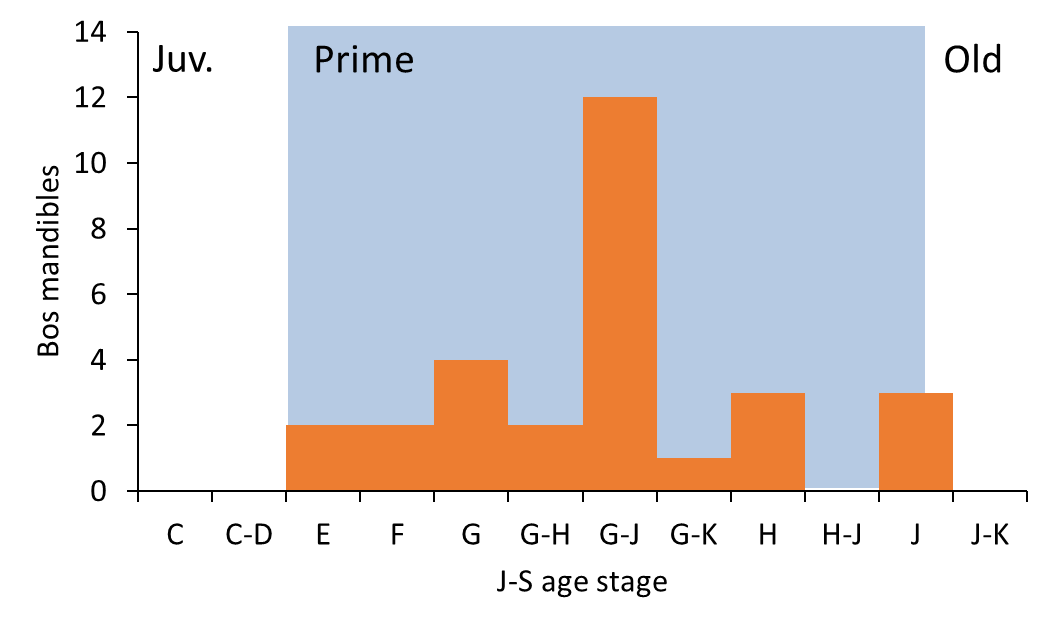


Supplementary Fig. S1. The distribution of aurochs dental age stages in NR Unit III, based on the Jones-Sadler (J-S) scheme. Data are from Supplementary Table S3. Plot prepared by Microsoft Excel.


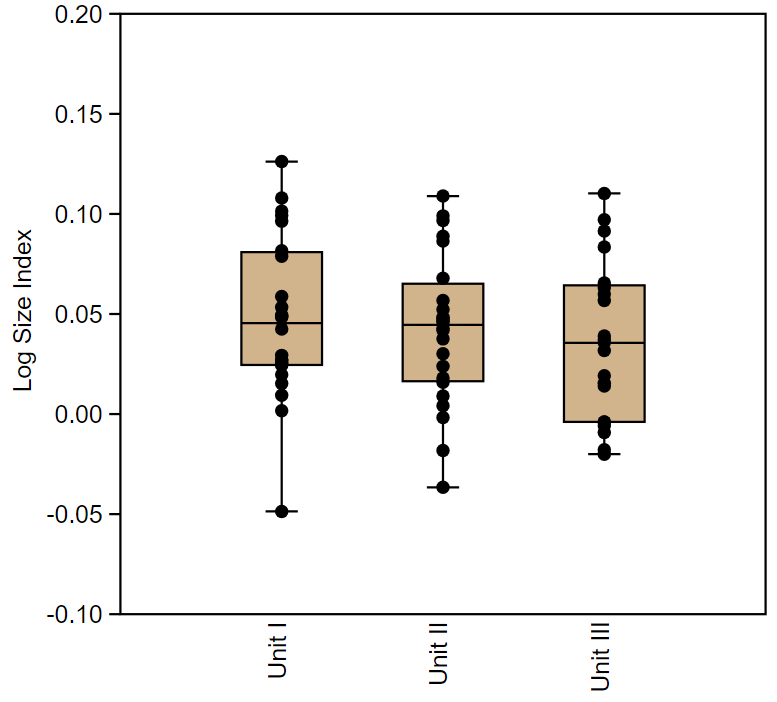


Supplementary Fig. S2. Aurochs LSI values: a comparison of bone width or breadth measurements among NR units I–III. The means and SD are statistically similar (ANOVA *F* = 0.503, *p* = 0.607). For each sample, each value is plotted as a dot. The box represents the 25–75% quartiles, the horizontal line inside the box represents the median, and the short vertical lines ("whiskers") show the minimal and maximal values. Data from Supplementary Table S4.

Supplementary Fig. S3. Microwear pattern on the analyzed teeth of aurochs (N=10) observed at ×35 magnification. The scale bar (0.4 mm) indicates the size of the analyzed area using an ocular reticle of 0.4 × 0.4 mm (0.16 mm²). All specimens correspond to left lower third molars (m3) to avoid replicating the same individuals. The images illustrate the general microwear pattern; however, due to lighting effects, not all features are simultaneously visible. These images are intended for qualitative illustration only, as quantitative measurements were performed directly under the stereomicroscope.


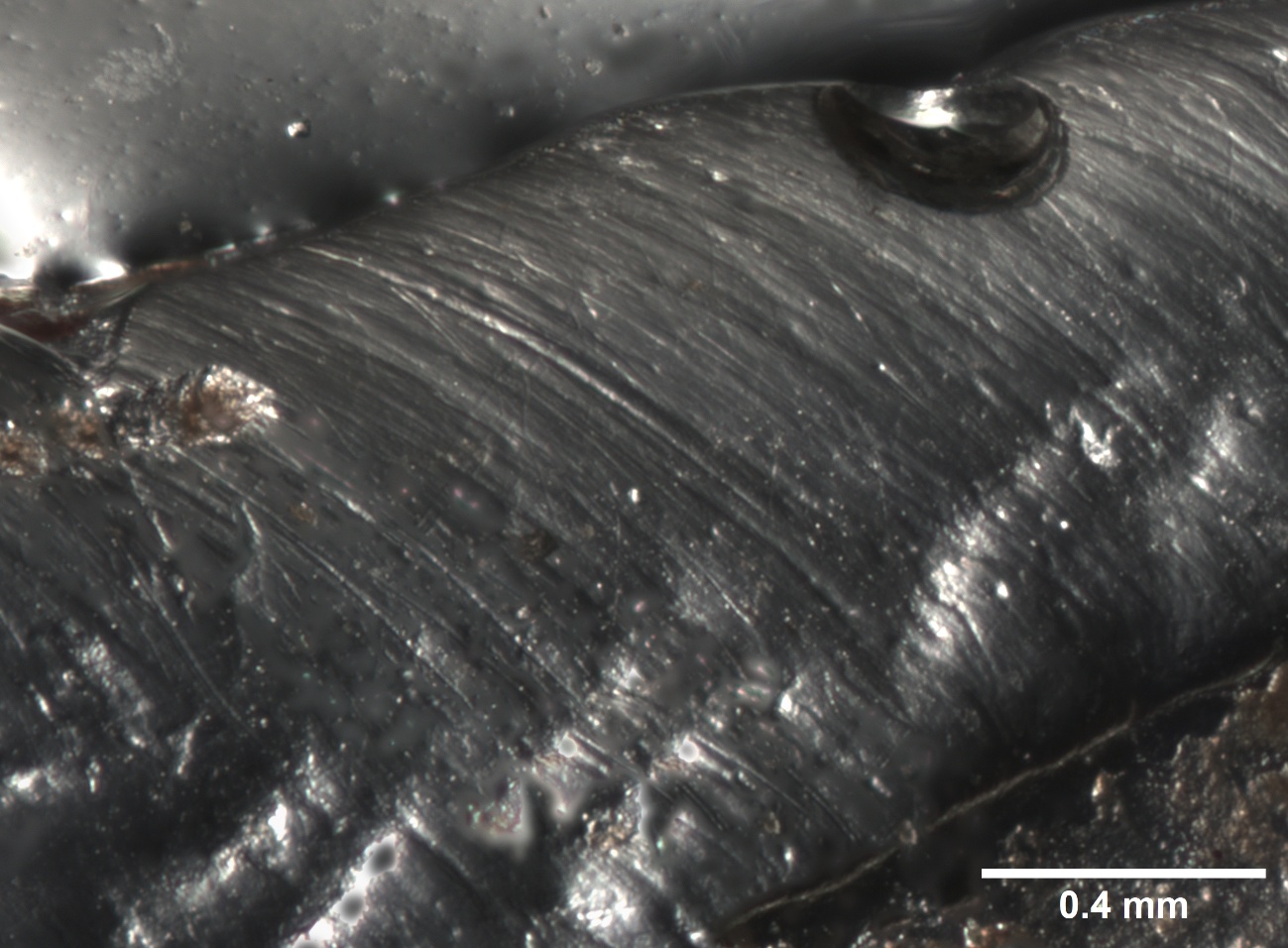


NR 322, with large pits and coarse scratches.


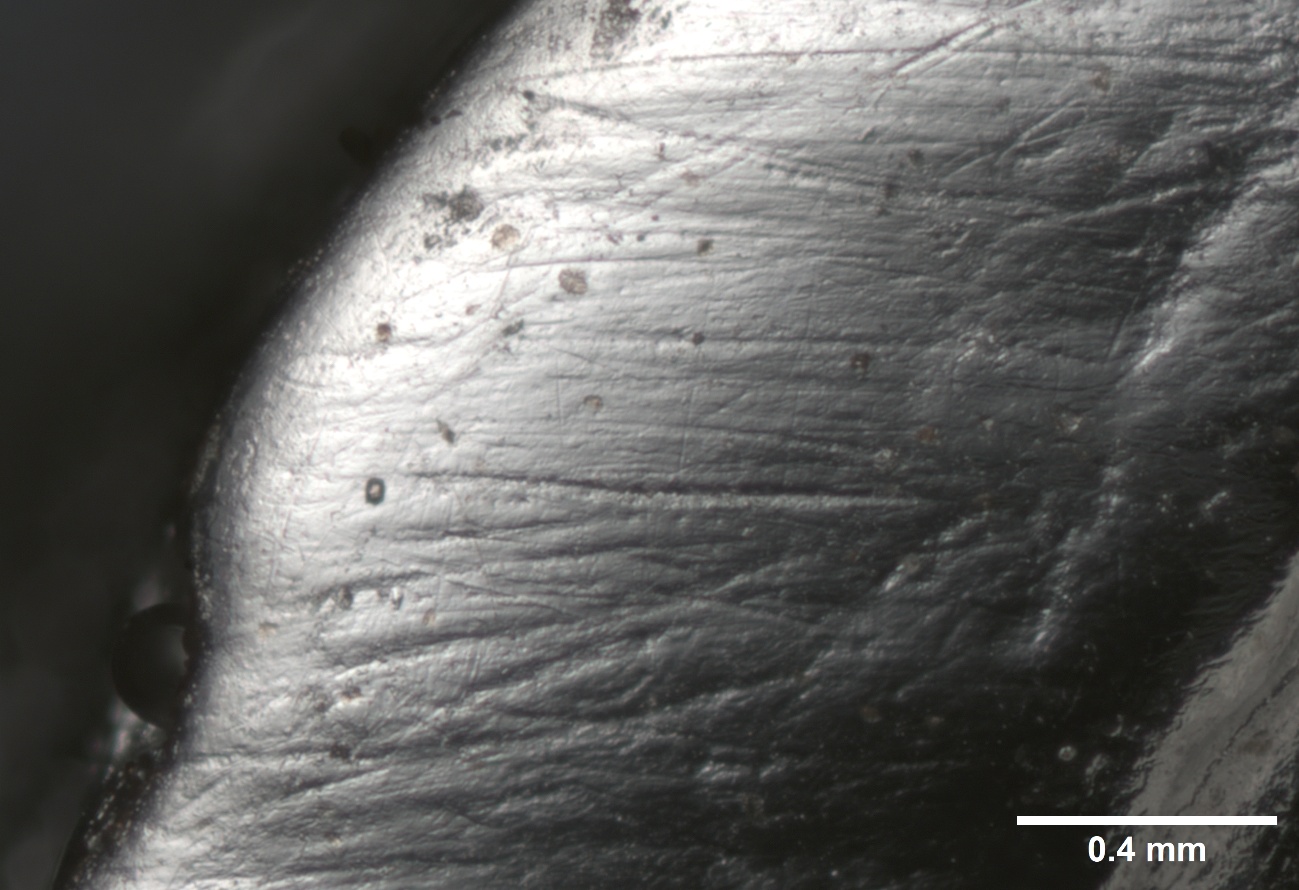


NR 1202, with large pits, and coarse + hypercoarse scratches.


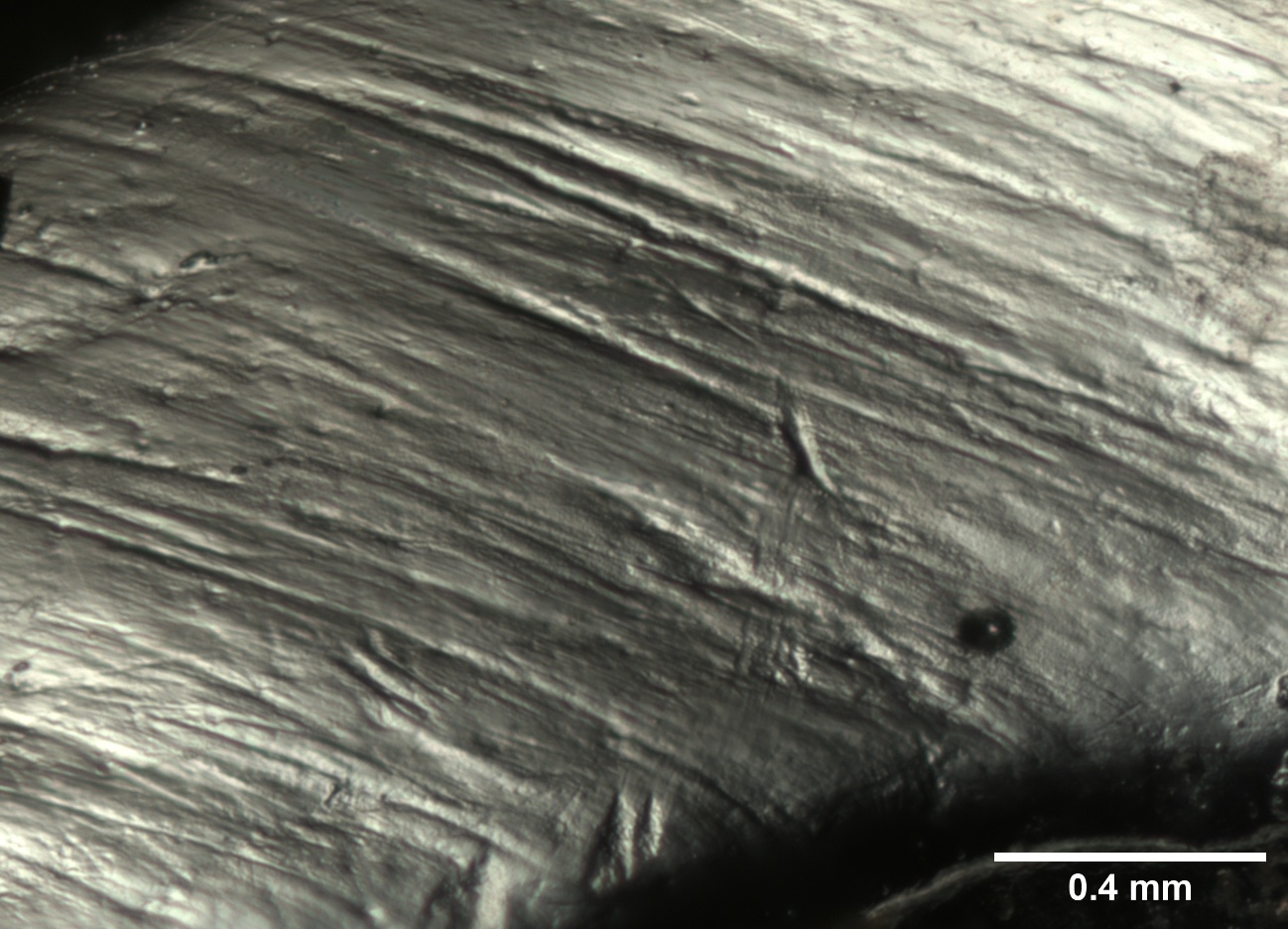


NR 1207, with large pits, and coarse + hypercoarse scratches.


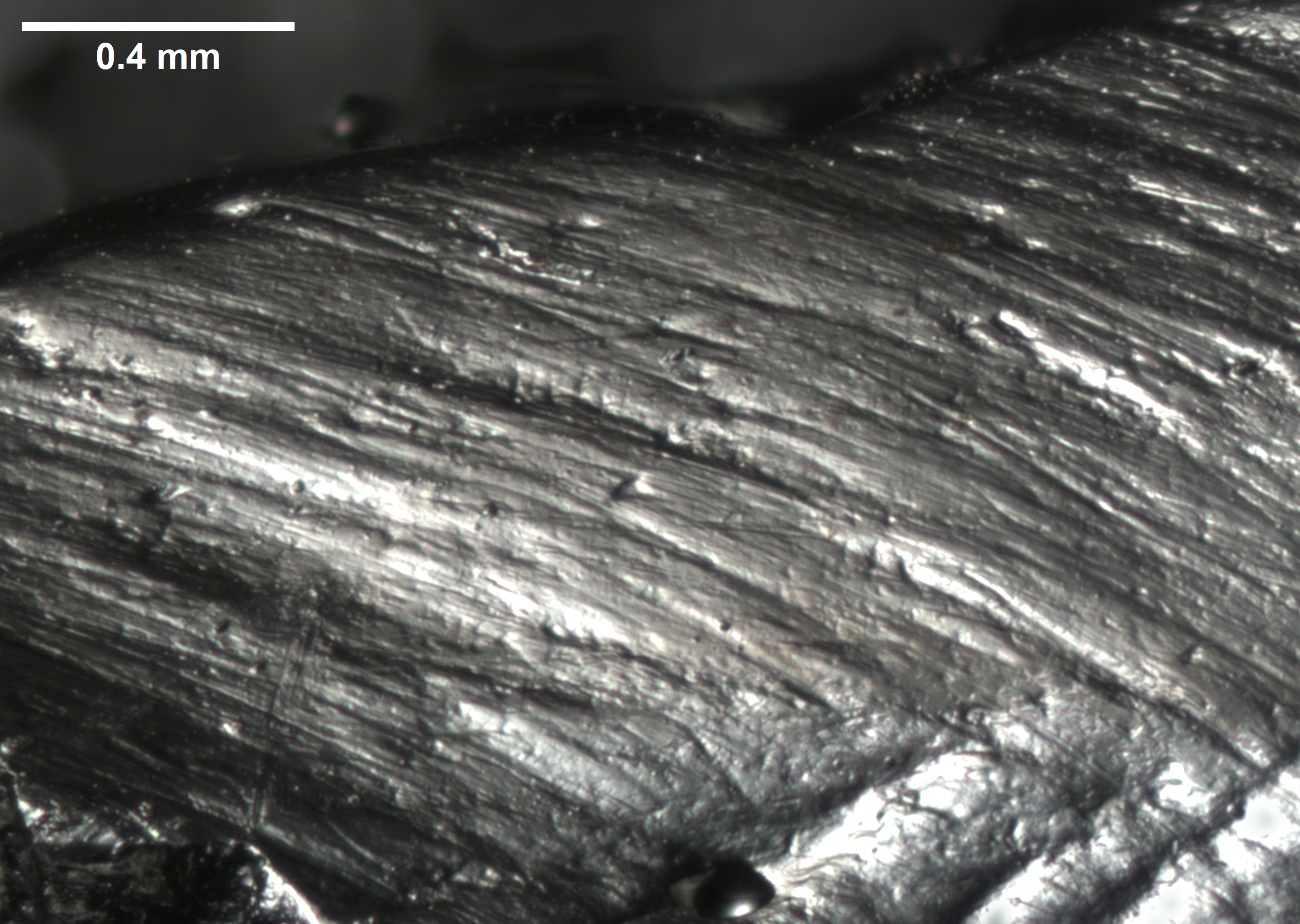


NR 1212, with large pits and fine + coarse scratches.


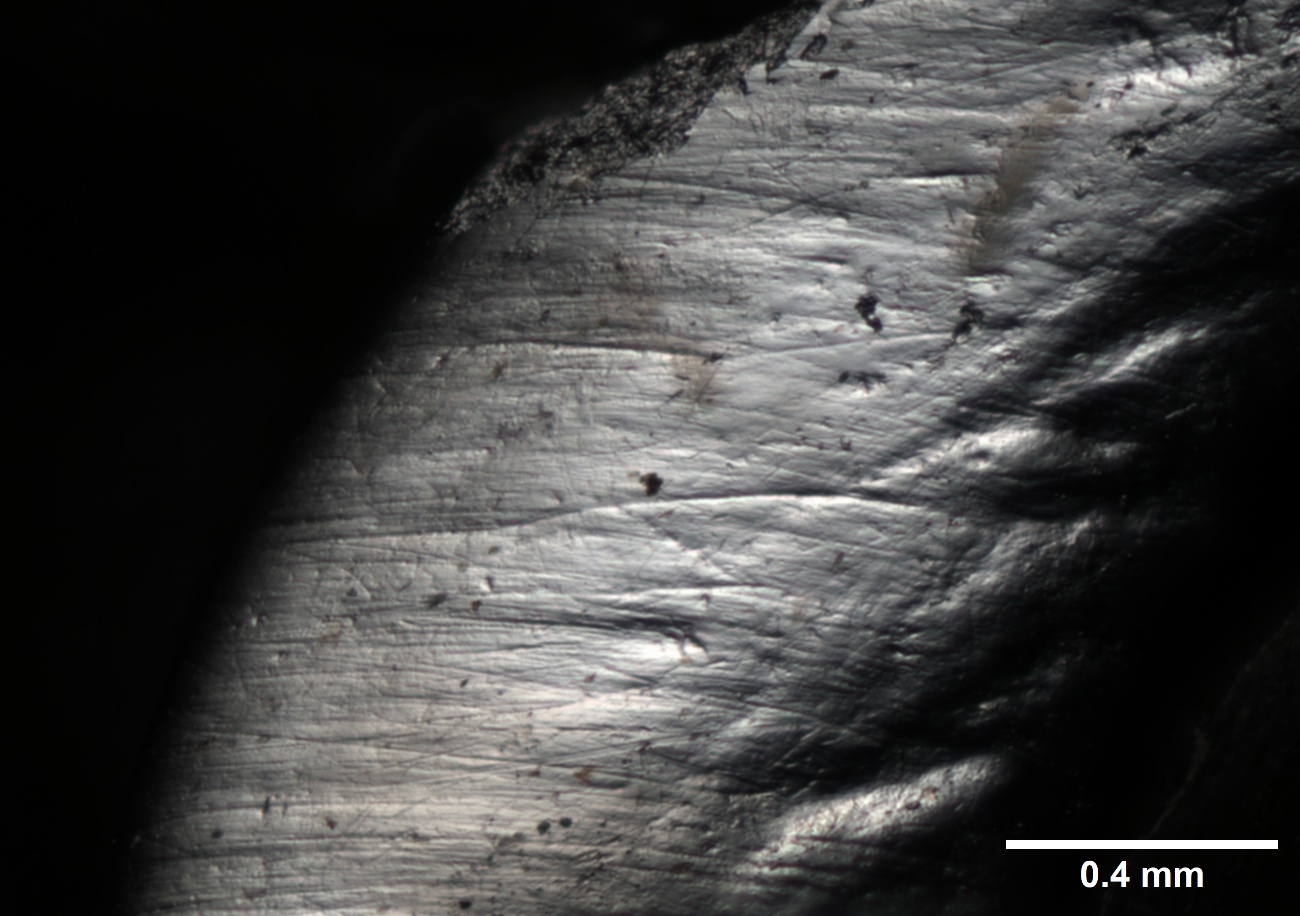


NR 1214, with large pits and fine + coarse scratches.


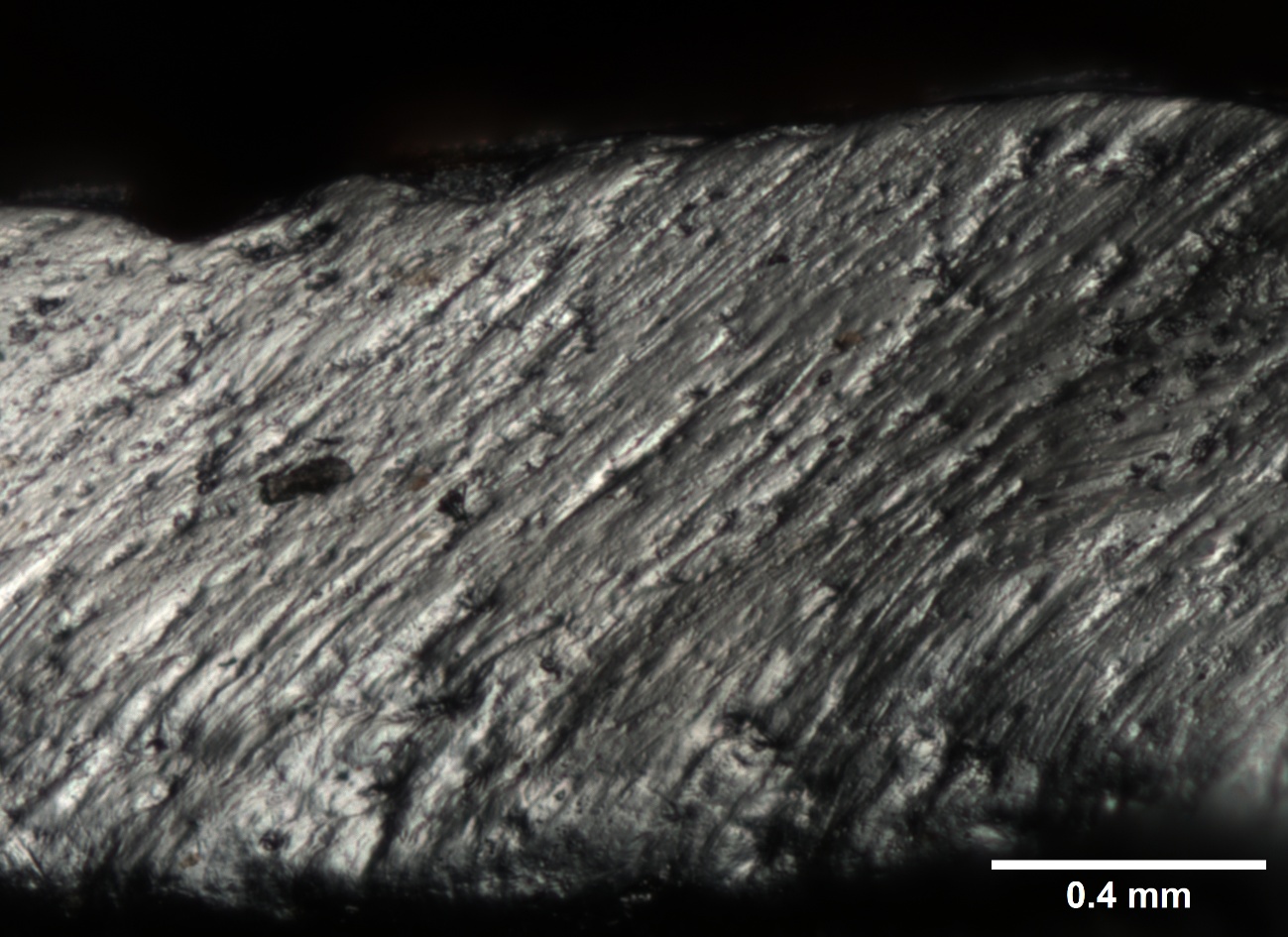


NR 1226, with large pits and coarse + hypercoarse scratches.


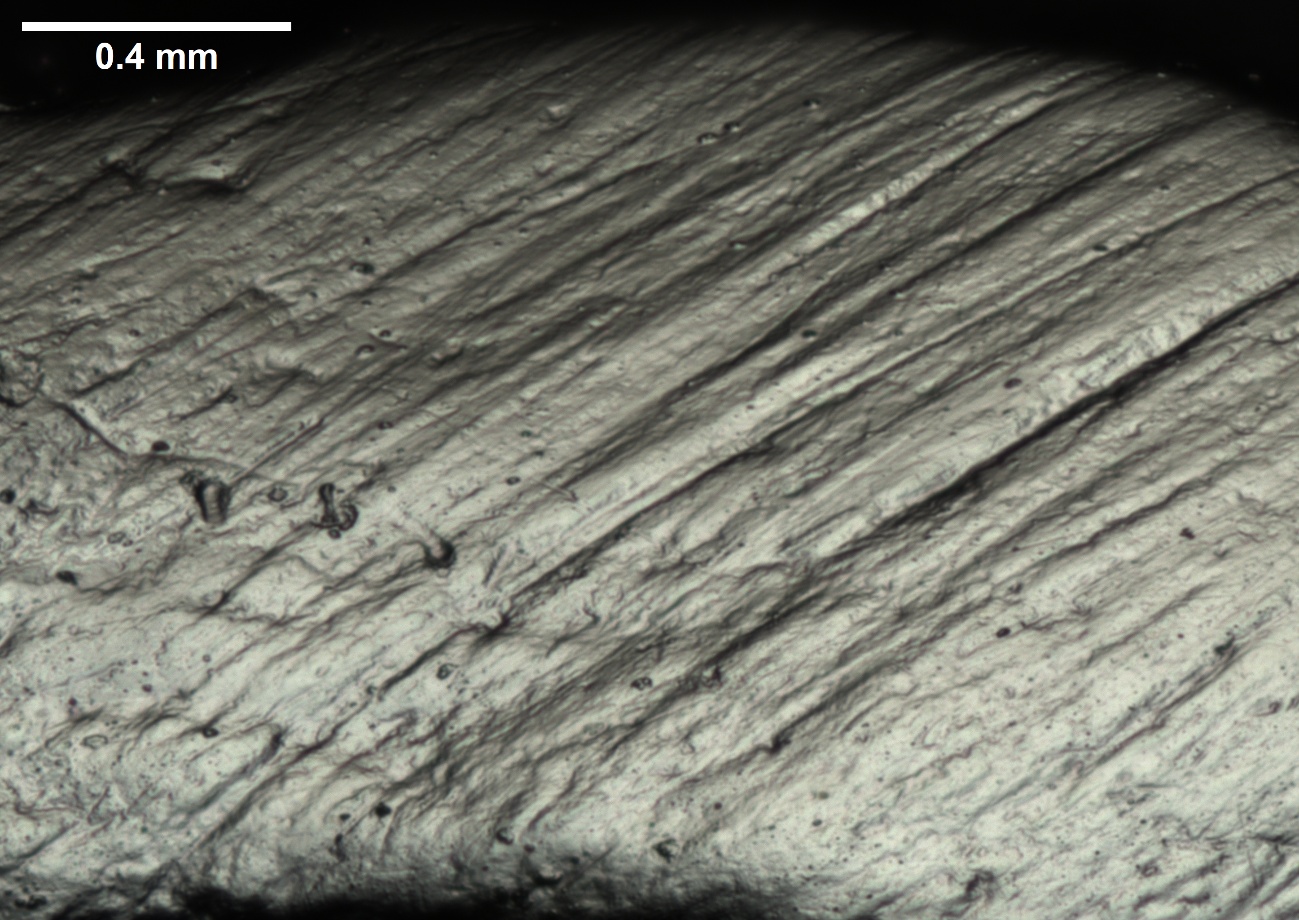


NR 1233, with large pits and coarse + hypercoarse scratches.


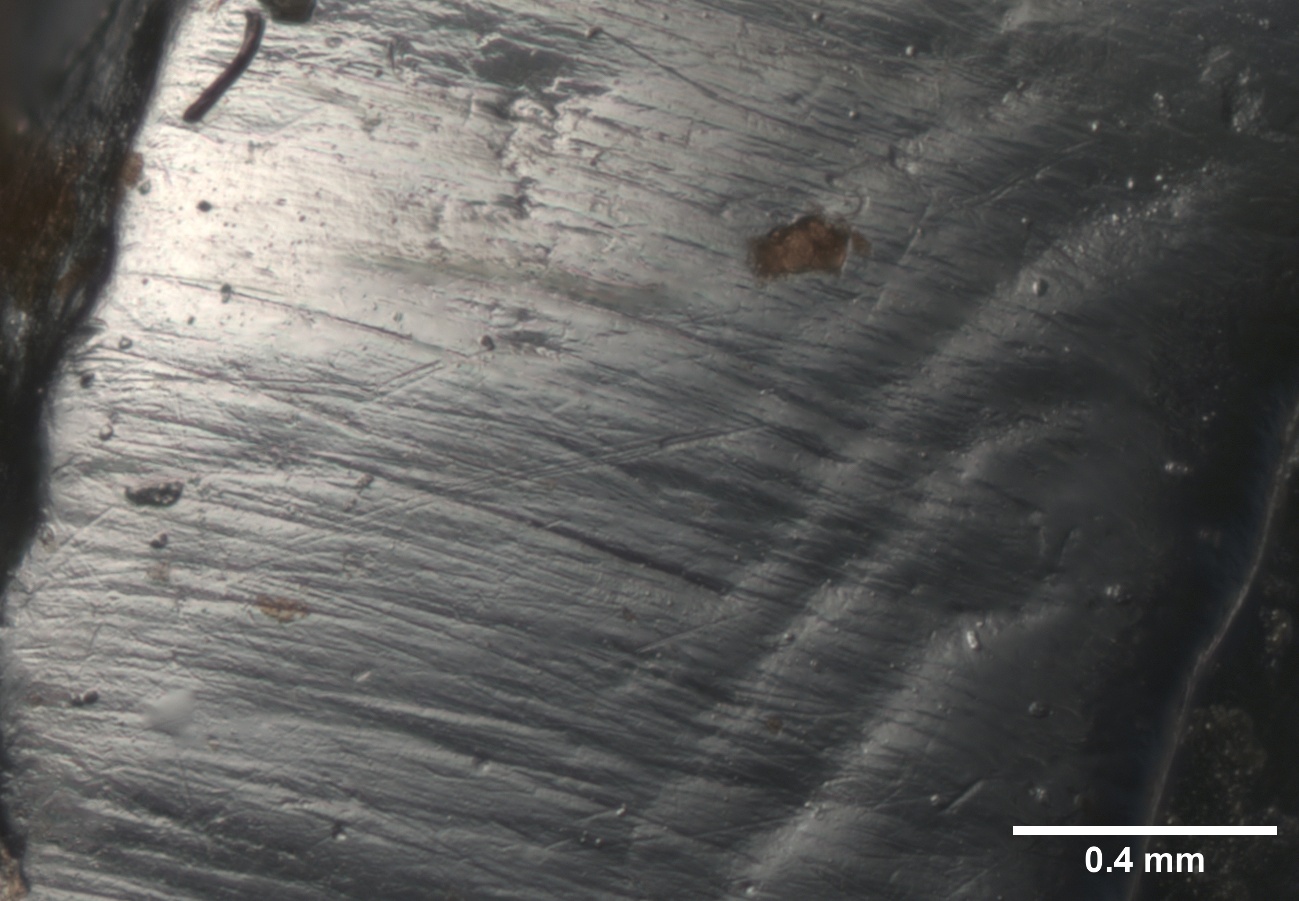


NR 1234, with large pits and fine + coarse scratches.


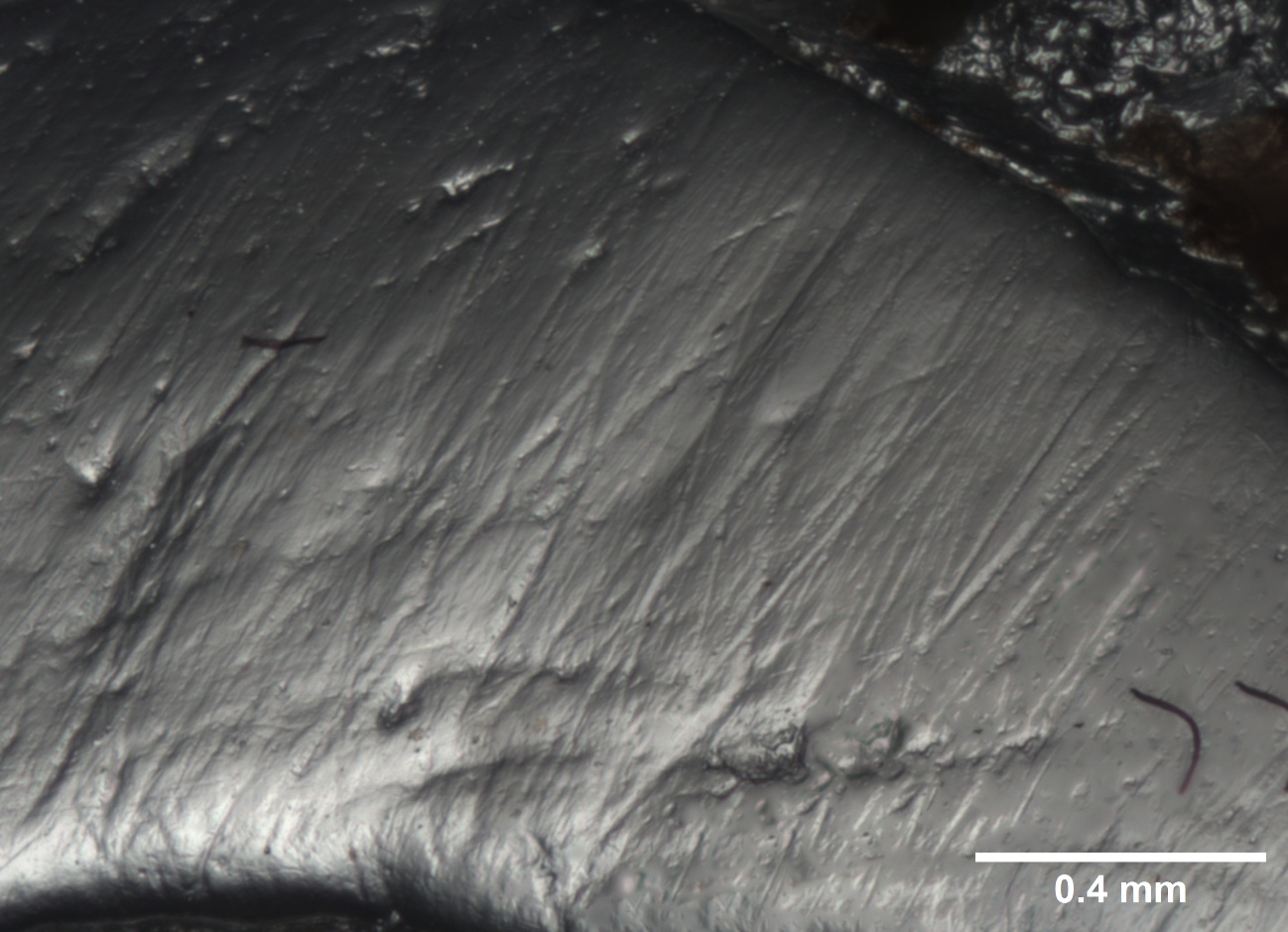


NR 1256, with large pits and fine + coarse scratches.


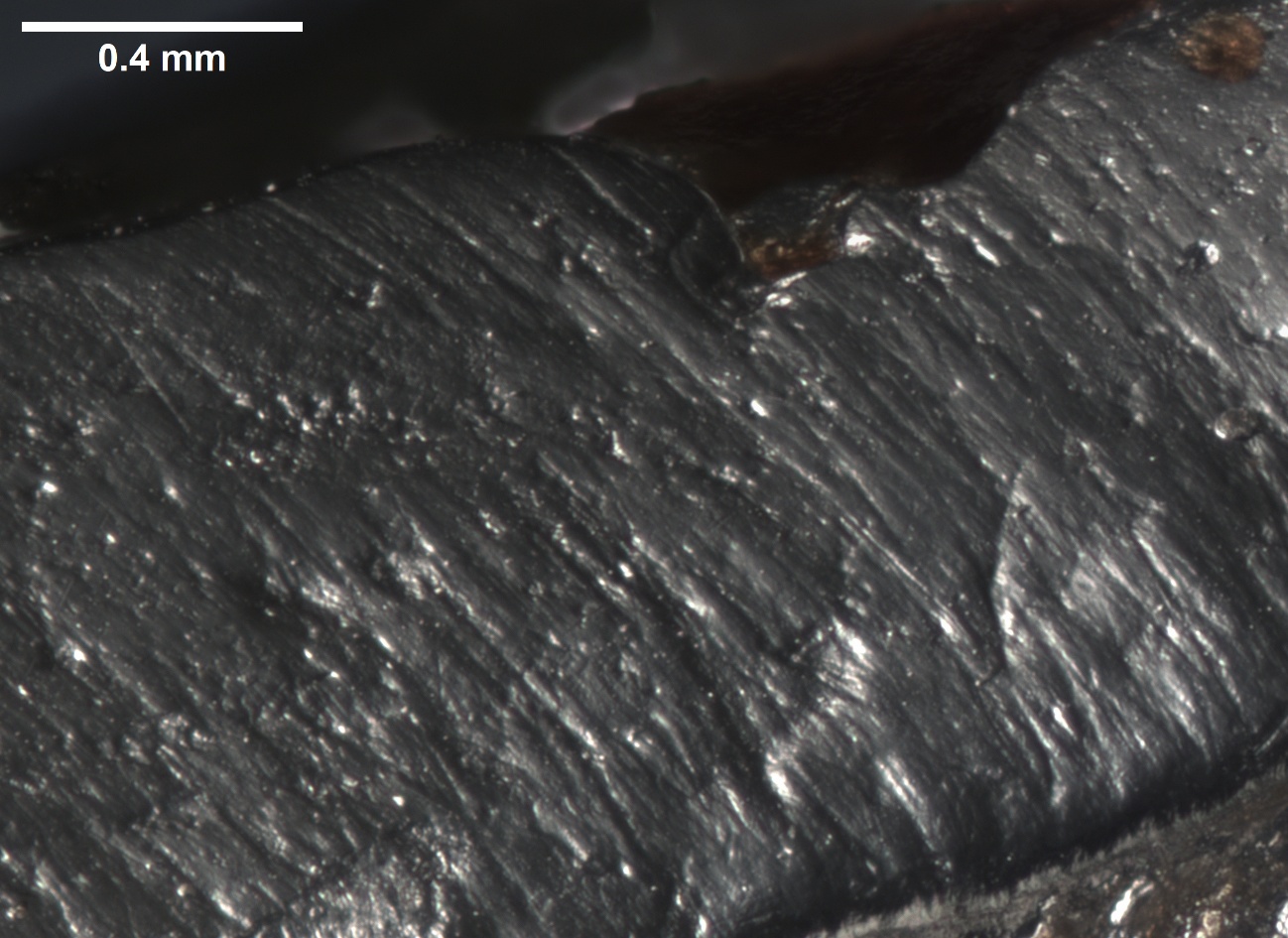


NR 1264, with large pits and coarse + hypercoarse scratches.


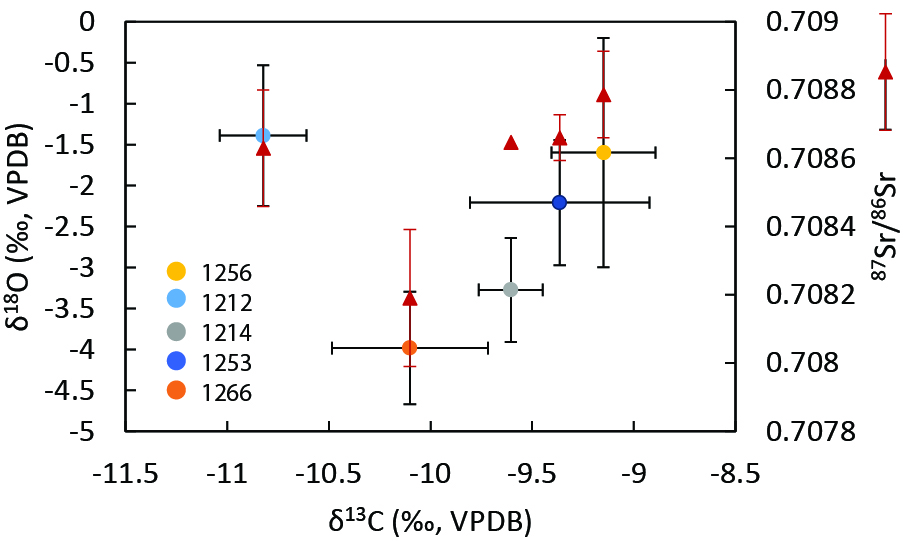


Supplementary Fig. S4. Bivariate plot of aurochs tooth enamel mean δ^13^C values against δ^18^O values ±1σ (color-filled circles and black error lines). Secondary plot of mean δ^13^C values against ^87^Sr/^86^Sr ratios (red triangles and error lines in red).

**Supplementary Table S1: Ungulate genera abundance data (NISP) for MP sites in the Mediterranean Zone of the southern Levant.**

|  | *Gazella* | *Dama* | *Capra* | *Cervus* | *Sus* | *Bos* | *Capreolus* | *Equus* | *Stephan-orhinus* | *Alcelaphus* | *Hippo.* | *Camelus* | Source |
| --- | --- | --- | --- | --- | --- | --- | --- | --- | --- | --- | --- | --- | --- |
| Misliya | 171 | 131 | 8 | 6 | 14 | 32 | 1 | 0 | 0 | 0 | 0 | 0 | 92 |
| Hayonim | 1912 | 1178 | 8 | 155 | 176 | 281 | 11 | 22 | 2 | 0 | 0 | 0 | 93 |
| Emanuel | 49 | 24 | 0 | 7 | 0 | 21 | 1 | 0 | 0 | 0 | 0 | 0 | 94 |
| Qafzeh | 91 | 193 | 97 | 198 | 62 | 171 | 5 | 78 | 35 | 7 | 0 | 0 | 95 |
| Geula | 221 | 309 | 13 | 44 | 118 | 209 | 38 | 21 | 22 | 5 | 1 | 0 | 96 |
| Amud | 291 | 60 | 18 | 5 | 17 | 1 | 1 | 0 | 1 | 0 | 0 | 0 | 97 |
| Kebara | 4573 | 2507 | 74 | 968 | 400 | 2030 | 20 | 133 | 17 | 12 | 0 | 0 | 98 |
| Shovakh | 27 | 5 | 4 | 0 | 1 | 2 | 0 | 0 | 0 | 0 | 0 | 0 | 99 |
| Tabun D | 187 | 141 | 6 | 46 | 13 | 31 | 5 | 3 | 12 | 0 | 7 | 0 | 35 |
| Tabun C | 187 | 43 | 15 | 21 | 38 | 100 | 0 | 15 | 26 | 0 | 35 | 0 | 35 |
| Tabun B | 332 | 1729 | 21 | 17 | 9 | 49 | 3 | 4 | 0 | 0 | 0 | 0 | 35 |
| Rantis | 14 | 75 | 3 | 0 | 2 | 4 | 0 | 0 | 0 | 0 | 0 | 0 | 100 |
| NR III | 38 | 6 | 0 | 2 | 1 | 229 | 0 | 82 | 0 | 0 | 0 | 0 | 31 |
| NR IIb | 3 | 12 | 0 | 2 | 0 | 9 | 0 | 1 | 0 | 0 | 0 | 0 | 34 |
| NR IIa | 12 | 30 | 2 | 10 | 0 | 13 | 0 | 3 | 5 | 0 | 0 | 0 | 34 |
| NR I | 46 | 31 | 124 | 2 | 4 | 65 | 2 | 50 | 2 | 0 | 0 | 0 | 34 |
| Quneitra | 11 | 8 | 1 | 7 | 0 | 43 | 0 | 28 | 1 | 0 | 0 | 0 | 101 |
| Fa’rah II | 1 | 0 | 1 | 0 | 0 | 24 | 0 | 49 | 0 | 26 | 0 | 4 | 102 |
| Ein Qashish | 21 | 23 | 2 | 1 | 4 | 68 | 1 | 4 | 0 | 0 | 0 | 0 | 37 |

**Supplementary Table S2. NR Unit III aurochs aging.** A) Fusion data for the late-fusing bone elements (skeletal elements that fuse at 30–40 months of age, roughly parallel to the P4 eruption). B) Raw data of the ageable dental specimens. Each row is a specimen, featuring its catalog number (CAT#), an indication whether it was sampled for microwear (MW) or isotope analysis (IS), the skeletal element (DN, mandible bone), its age stage, age “number”, and age category according to Jones and Sadler [71], its interpretation in a 3-cohort system (juvenile, prime, old) and a 2-cohort system (juvenile or adult), and for each tooth present, crown height measurements of anterior and posterior cusps (AC and PC, respectively), and occlusal wear stage (WS) according to [71]. C) Counts of J-S stages for aurochs mandibular aging, based on the former data. D) Summary of the three aging schemes.

A)

|  | fused | unfused |
| --- | --- | --- |
| Calcaneus-tuber calcis | 1 | 0 |
| Humerus-proximal | 1 | 0 |
| Femur-proximal | 1 | 0 |
| Femur-distal | 6 | 0 |
| Metapodial-distal | 6 | 1 |
| Radius-distal | 0 | 1 |
| Tibia-proximal | 0 | 0 |
| Tibia-distal | 8 | 0 |
| Ulna-proximal | 0 | 0 |
| Ulna-distal | 1 | 0 |

B)

| CAT# | SAMPLED | SIDE | BONE | J-S stage | J-S number | Age in y | triplot | biplot | M3 |  |  | M2 |  |  | M1 |  |  | P4 |  |
| --- | --- | --- | --- | --- | --- | --- | --- | --- | --- | --- | --- | --- | --- | --- | --- | --- | --- | --- | --- |
|  |  |  |  |  |  |  |  |  | AC | PC | WS | AC | PC | WS | AC | PC | WS | AC | WS |
| 1334 | MW | R | DN + M3 | ? |  |  |  |  |  |  | q |  |  |  |  |  |  |  |  |
| 343 |  | L | M3 | E | 17 | adult2 | Prime | Adult |  | 50.5 | C |  |  |  |  |  |  |  |  |
| 1266 | MW IS | R | M3 | E | 17 | adult2 | Prime | Adult |  | 77 | C |  |  |  |  |  |  |  |  |
| 1226 | MW | L | DN + M3 | F | 21 | adult3 | Prime | Adult | 54.7 | 51.4 | F q |  |  |  |  |  |  |  |  |
| 1264 | MW IS | L | M3 | F | 19 | adult3 | Prime | Adult |  | 51.7 | F |  |  |  |  |  |  |  |  |
| 22 |  | R | M3 | G | 20-22 | adult3 | Prime | Adult | 57.5 | 61.3 | G |  |  |  |  |  |  |  |  |
| 322 |  | L | DN+M3-P2 | G | 22 | adult3 | Prime | Adult |  |  | K q |  |  | K q |  |  | ERUPTED |  | G |
| 132 |  | R | DN+M2-P2 | G | 22 | adult3 | Prime | Adult |  |  |  |  |  | K q | 13.86 | 17.7 | J z | 17.9 | J |
| 1311 | MW | R | DN + M2-P2 | G | 22 | adult3 | Prime | Adult |  |  |  | 33.8 |  | K q |  |  | K |  |  |
| 4350 |  | R | DN+M3 | G-H |  | adult3/elderly | Prime | Adult | 27.67 |  | K q |  |  |  |  |  |  |  |  |
| 1243 | MW | R | DN + M3 | G-H |  | adult3/elderly | Prime | Adult |  |  | K q |  |  |  |  |  |  |  |  |
| 592 |  | L | M3 | G-J |  | adult3/elderly | Old | Adult | 23 | 28 | K |  |  |  |  |  |  |  |  |
| 632 |  | R | M3 | G-J |  | adult3/elderly | Prime | Adult | 27.6 | 29.7 | K |  |  |  |  |  |  |  |  |
| 1215 | MW | R | M3 | G-J |  | adult3/elderly | Prime | Adult | 34.6 | 34.6 | J |  |  |  |  |  |  |  |  |
| 1324 | MW IS | R | M3 | G-J |  | adult3/elderly | Prime | Adult |  | 35.1 | K |  |  |  |  |  |  |  |  |
| 1214 | MW IS | L | M3 | G-J |  | adult3/elderly | Prime | Adult |  | 37.5 | K |  |  |  |  |  |  |  |  |
| 1256 | MW IS | L | M3 | G-J |  | adult3/elderly | Prime | Adult | 38.2 | 39.8 | J |  |  |  |  |  |  |  |  |
| 1212 | MW IS | L | M3 | G-J |  | adult3/elderly | Prime | Adult |  | 43.7 | K |  |  |  |  |  |  |  |  |
| 6 |  | R | M3 | G-J |  | adult3/elderly | Prime | Adult | 46.4 | 48.4 | H |  |  |  |  |  |  |  |  |
| 1233 | MW IS | L | M3 | G-J |  | adult3/elderly | Prime | Adult |  | 50.7 | J |  |  |  |  |  |  |  |  |
| 1253 | MW IS | R | M3 | G-J |  | adult3/elderly | Prime | Adult |  | 55.5 | J |  |  |  |  |  |  |  |  |
| 1273 | MW IS | R | M3 | G-J |  | adult3/elderly | Prime | Adult | 31.2 |  | K |  |  |  |  |  |  |  |  |
| 21 |  | R | DN + M3 | G-J |  | adult3/elderly |  | Adult |  |  | K |  |  |  |  |  |  |  |  |
| 4346 |  | L | DN+M2-P4 | G-K |  | adult3/elderly |  | Adult |  |  |  |  |  | K |  |  | J z |  | G |
| 4348 |  | R | DN+M2-M1 | H | 25 | elderly | Prime | Adult |  |  |  | 15.95 |  | J z | 23.31 | 21.28 | K y |  |  |
| 1300 | MW | R | DN + M3-M1 | H | 24 | elderly | Prime | Adult |  |  | J q |  |  | K y | 13.3 | 13.2 | K z |  |  |
| 4200 |  | R | DN+M2 | H | 24 | elderly | Prime | Adult |  |  |  |  |  | K y |  |  |  |  |  |
| 759 |  | L | DN + M3 | J | 26 | elderly | Old | Adult |  | 22.9 | G x |  |  |  |  |  |  |  |  |
| 1202 | MW | L | DN + M3 | J | 27 | elderly | Old | Adult | 23.5 | 23.9 | K y |  |  |  |  |  |  |  |  |
| 1207 | MW | L | DN +M3-M2 | J | 26 | elderly |  | Adult |  |  | K x |  | 26 | K x |  |  |  |  |  |
| 5 |  | L | M3 |  |  |  | Old | Adult |  | 8.1 | G-M (VERY WORN) |  |  |  |  |  |  |  |  |
| 1213 | MW | L | P4 |  |  |  |  | Adult |  |  |  |  |  |  |  |  |  | 23.3 | E |
| 1262 | MW | R | DN + M1-P3 |  |  |  |  |  |  |  |  |  |  |  |  |  | L z |  |  |
| 4345 |  | R | DN+P4-P3 |  |  |  |  | Adult |  |  |  |  |  |  |  |  |  |  | C |
| 4347 |  | L | DN+M1-P3 |  |  |  |  | Adult |  |  |  |  |  |  |  |  | K |  | F |
| 4349 |  | R | P4 |  |  |  |  | Adult |  |  |  |  |  |  |  |  |  | 34.5 | E |

C)

| J-S Stage | N mandibles | Age cohort |
| --- | --- | --- |
| C |  | Juvenile |
| C-D |  |  |
| E | 2 | Prime |
| F | 2 |  |
| G | 4 |  |
| G-H | 2 |  |
| G-J | 12 |  |
| G-K | 1 |  |
| H | 3 |  |
| H-J |  |  |
| J | 3 | Prime or Old |
| J-K |  | Old |

D)

|  | Juvenile | Adult+Old |
| --- | --- | --- |
| Bone fusion (n=26) | 2 | 24 |
| Dental, J-S aging (n=29) | 0 | 29 |
| Dental, dP_4_-M_3_ (n=23) | 0 | 23 |

**Supplementary Table S3: Data for the triangular aging plot.**

|  | Juvenile | Prime | Old | Reference |
| --- | --- | --- | --- | --- |
| NR III | 0 | 23 | 4 | This study |
| Avetrana | 15 | 35 | 3 | 39 |
| Kebara MP | 22 | 108 | 43 | 93 |
| Mauran | 36 | 103 | 6 | 25 |

**Supplementary Table S4: Raw measurements and mixture analysis results for aurochs elements in NR Units I–III.** For each specimen (row), the raw measurement is given, following von den Driesch’s [76] codes. Then, the measurement from standard animal (76,77), and the LSI value are given. LSI values can be assigned to the group that shows the largest value. Group 1's mean LSI value is 0.030125 (SD=0.030506). Group 2 contains larger specimens: mean LSI value 0.099295 (SD=0.011235). Therefore, groups 1 and 2 were interpreted as female and male animals, respectively.

Bone abbreviations are: AS, astragalus; HU, humerus; MC, metacarpus; MR, metatarsus; NC, naviculo-cuboid; RA, radius; TI, tibia.

| Unit | Element | **Measurement (mm)** | Standard (mm) | LSI | Group1 | Group2 | Max group |
| --- | --- | --- | --- | --- | --- | --- | --- |
| IIB | AS-GLl | 79.6 | 83 | -0.018165025 | 3.077 | 1.16E-23 | 1 |
| IIB | AS-GLl | 90.5 | 83 | 0.037570487 | 10.45 | 1.75E-06 | 1 |
| IIB | AS-GLl | 91.4 | 83 | 0.041868103 | 10 | 1.33E-05 | 1 |
| IIB | AS-GLl | 92.3 | 83 | 0.046123609 | 9.386 | 8.58E-05 | 1 |
| IIB | HU-BT | 108.6 | 89 | 0.086439819 | 1.96 | 3.256 | 2 |
| IIB | HU-BT | 111.2 | 89 | 0.096714781 | 0.9944 | 6.103 | 2 |
| IIB | HU-BT | 99.1 | 89 | 0.046683648 | 9.294 | 0.000109 | 1 |
| IIB | MC-Bd | 76.1 | 73 | 0.018061797 | 9.96 | 2.79E-11 | 1 |
| IIB | MC-Bd | 91.7 | 73 | 0.099046476 | 0.8391 | 6.264 | 2 |
| IIB | MC-Bp | 81.7 | 74 | 0.042990337 | 9.853 | 2.21E-05 | 1 |
| IIB | MC-Bd | 90.8 | 74 | 0.088854129 | 1.688 | 4.069 | 2 |
| IIB | MR-Bd | 75.9 | 68 | 0.047732863 | 9.117 | 0.000167 | 1 |
| IIB | MR-Bp | 69.3 | 62 | 0.048341545 | 9.011 | 0.000214 | 1 |
| IIB | NC-GB | 68.4 | 67 | 0.008981299 | 8.47 | 5.83E-14 | 1 |
| IIB | NC-GB | 86.1 | 67 | 0.108928349 | 0.383 | 4.339 | 2 |
| IIB | TI-Bd | 71.7 | 78 | -0.036575447 | 0.9866 | 1.10E-31 | 1 |
| IIB | TI-Bd | 77.7 | 78 | -0.001673584 | 6.256 | 1.82E-17 | 1 |
| IIB | TI-Bd | 80.9 | 78 | 0.015853919 | 9.653 | 6.61E-12 | 1 |
| IIB | TI-Bd | 82.4 | 78 | 0.023832609 | 10.54 | 1.00E-09 | 1 |
| IIB | TI-Bd | 83.6 | 78 | 0.030111675 | 10.77 | 3.66E-08 | 1 |
| IIB | TI-Bd | 88.9 | 78 | 0.056807158 | 7.347 | 0.004915 | 1 |
| IIB | TI-Bd | 91.2 | 78 | 0.067900236 | 5.003 | 0.1263 | 1 |
| III | AS-GLl | 90.4 | 83 | 0.037090338 | 10.49 | 1.38E-06 | 1 |
| III | AS-GLl | 95.3 | 83 | 0.060014808 | 6.664 | 0.01389 | 1 |
| III | HU-BT | 87.8 | 89 | -0.005895491 | 5.364 | 5.79E-19 | 1 |
| III | HU-BT | 88.2 | 89 | -0.003921422 | 5.777 | 2.95E-18 | 1 |
| III | MC-Bd | 91.3 | 73 | 0.097147917 | 0.964 | 6.153 | 2 |
| III | MC-Bd | 94.1 | 73 | 0.110266763 | 0.3417 | 3.89 | 2 |
| III | MR-Bd | 77.5 | 68 | 0.05679279 | 7.35 | 0.004892 | 1 |
| III | MR-Bp | 59.2 | 62 | -0.020069983 | 2.782 | 1.94E-24 | 1 |
| III | MR-Bp | 59.5 | 62 | -0.017874724 | 3.123 | 1.51E-23 | 1 |
| III | MR-Bp | 64.8 | 62 | 0.019183316 | 10.1 | 5.71E-11 | 1 |
| III | MR-Bp | 66.7 | 62 | 0.031734144 | 10.75 | 8.81E-08 | 1 |
| III | MR-Bp | 67.3 | 62 | 0.035623375 | 10.6 | 6.65E-07 | 1 |
| III | MR-Bp | 71.7 | 62 | 0.063127466 | 5.999 | 0.03522 | 1 |
| III | NC-GB | 82.7 | 67 | 0.091430707 | 1.43 | 4.905 | 2 |
| III | NC-GB | 69.2 | 67 | 0.014031292 | 9.371 | 1.95E-12 | 1 |
| III | NC-GB | 81.2 | 67 | 0.083481227 | 2.333 | 2.327 | 1 |
| III | RA-Bp | 103.6 | 100 | 0.015359755 | 9.579 | 4.76E-12 | 1 |
| III | RA-Bp | 109.4 | 100 | 0.039017322 | 10.32 | 3.52E-06 | 1 |
| III | RA-Bp | 97.9 | 100 | -0.009217308 | 4.689 | 3.48E-20 | 1 |
| III | TI-Bd | 77.3 | 78 | -0.003915109 | 5.779 | 2.97E-18 | 1 |
| III | TI-Bd | 90.7 | 78 | 0.065512684 | 5.495 | 0.0682 | 1 |
| I | AS-GLl | 87.8 | 83 | 0.024416424 | 10.58 | 1.42E-09 | 1 |
| I | AS-GLl | 87.9 | 83 | 0.024910783 | 10.61 | 1.90E-09 | 1 |
| I | AS-GLl | 88.8 | 83 | 0.029334873 | 10.77 | 2.38E-08 | 1 |
| I | AS-GLl | 93 | 83 | 0.049404856 | 8.82 | 0.000328 | 1 |
| I | HU-BT | 107.4 | 89 | 0.081614275 | 2.592 | 1.816 | 1 |
| I | HU-BT | 119 | 89 | 0.126156955 | 0.07593 | 0.3595 | 2 |
| I | HU-BT | 94.5 | 89 | 0.026041802 | 10.67 | 3.68E-09 | 1 |
| I | MC-Bd | 92.2 | 73 | 0.101408061 | 0.7024 | 6.156 | 2 |
| I | MC-Bd | 81.6 | 73 | 0.048367299 | 9.006 | 0.000216 | 1 |
| I | MC-Bd | 80.5 | 73 | 0.04247302 | 9.923 | 1.75E-05 | 1 |
| I | MC-Bd | 77.7 | 73 | 0.027098159 | 10.72 | 6.77E-09 | 1 |
| I | MR-Bd | 76.9 | 68 | 0.053417427 | 8.047 | 0.001501 | 1 |
| I | MR-Bd | 84.9 | 68 | 0.096398778 | 1.017 | 6.061 | 2 |
| I | NC-GB | 59.9 | 67 | -0.04864798 | 0.384 | 1.40E-37 | 1 |
| I | NC-GB | 70.1 | 67 | 0.019643215 | 10.15 | 7.64E-11 | 1 |
| I | NC-GB | 71.3 | 67 | 0.027014727 | 10.71 | 6.45E-09 | 1 |
| I | NC-GB | 75 | 67 | 0.048986461 | 8.896 | 0.000277 | 1 |
| I | NC-GB | 84.2 | 67 | 0.099237289 | 0.8274 | 6.266 | 2 |
| I | NC-GB | 85.9 | 67 | 0.107918361 | 0.417 | 4.667 | 2 |
| I | RA-Bp | 119.9 | 100 | 0.078819183 | 3.013 | 1.191 | 1 |
| I | TI-Bd | 78.3 | 78 | 0.001667159 | 6.97 | 2.52E-16 | 1 |
| I | TI-Bd | 79.7 | 78 | 0.009363719 | 8.543 | 7.66E-14 | 1 |
| I | TI-Bd | 80.8 | 78 | 0.015316758 | 9.573 | 4.63E-12 | 1 |
| I | TI-Bd | 89.3 | 78 | 0.058756856 | 6.933 | 0.009333 | 1 |
| IIA | AS-GLl | 83.8 | 83 | 0.004165926 | 7.498 | 1.70E-15 | 1 |
| IIA | AS-GLl | 93.6 | 83 | 0.052197756 | 8.289 | 0.000958 | 1 |

**Supplementary Table S5: Mesowear and microwear data for the aurochs from Unit III of Nesher Ramla.** N = Number of specimens; M = Mean; SD = Standard deviation; CV = Coefficient of variation; MWS = Mesowear score; Npit = Average number of pits; Nscr = Average number of scratches; %LP = Percentage of individuals with large pits; %G = Percentage of individuals with gouges; SWS = Scratch width score; %XS = Percentage of individuals with cross scratches, %HC = Percentage of individuals with hyper-coarse scratches.

|  | Mesowear | |  | Microwear | |  |  |  |  |  |  |
| --- | --- | --- | --- | --- | --- | --- | --- | --- | --- | --- | --- |
|  | N | MWS |  | N | Npit | Nscr | %LP | %G | SWS | %XS | %HC |
| M | 8 | 2.50 |  | 10 | 15.35 | 15.10 | 100 | 40 | 1.60 | 0 | 60 |
| SD |  | 0.53 |  |  | 4.01 | 1.54 |  |  |  |  |  |
| CV |  | 0.21 |  |  | 0.26 | 0.10 |  |  |  |  |  |

**Supplementary Table S6: Stable isotope data**

| **Cat#** | **Unit** | **n** | **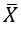**   \| **δ^13^C ±1σ** \| \| --- \| | **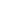**   \| **δ^13^C _min_** \| \| --- \| | **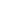**   \| **δ^13^C _max_** \| \| --- \| | **Δ^13^C_min-max_** | **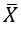**   \| **δ^18^O ±1σ** \| \| --- \| | **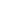**   \| **δ^18^O _min_** \| \| --- \| | **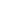**   \| **δ^18^O _max_** \| \| --- \| | **Δ^18^O_min-max_** | **n Sr** | **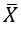**   \| **^87^Sr/^86^Sr ±1σ** \| \| --- \| |
| --- | --- | --- | --- | --- | --- | --- | --- | --- | --- | --- | --- | --- | --- | --- | --- | --- | --- | --- | --- |
| 1253 | III | 25 | -9.36±0.44 | -10.11 | -8.47 | 1.64 | -2.21±0.77 | -3.66 | -0.49 | 3.17 | 4 | 0.7087±0.00007 |
| 1266 | III | 26 | -10.10±0.38 | -10.80 | -8.97 | 1.83 | -3.98±0.69 | -5.25 | -1.89 | 3.38 | 3 | 0.7082±0.0002 |
| 1214 | III | 23 | -9.61±0.16 | -10.27 | -8.89 | 1.37 | -3.27±0.64 | -5.15 | -0.48 | 4.67 | 1 | 0.7086 |
| 1256 | III | 29 | -9.15±0.26 | -9.50 | -8.18 | 1.32 | -1.60±1.40 | -3.27 | 1.39 | 4.65 | 4 | 0.7088±0.0001 |
| 1212 | III | 27 | -10.82±0.21 | -11.22 | -11.22 | 0.84 | -1.39±0.86 | -3.28 | 0.15 | 3.43 | 4 | 0.7086±0.0002 |
